# Oxidation by Neutrophils-Derived HOCl Increases Immunogenicity of Proteins by Converting Them into Ligands of Several Endocytic Receptors Involved in Antigen Uptake by Dendritic Cells and Macrophages

**DOI:** 10.1371/journal.pone.0123293

**Published:** 2015-04-07

**Authors:** Rafał Biedroń, Maciej K. Konopiński, Janusz Marcinkiewicz, Szczepan Józefowski

**Affiliations:** 1 Department of Immunology, Jagiellonian University Medical College, Cracow, Poland; 2 Institute of Nature Conservation, Polish Academy of Sciences, Cracow, Poland; INSERM, FRANCE

## Abstract

The initiation of adaptive immune responses to protein antigens has to be preceded by their uptake by antigen presenting cells and intracellular proteolytic processing. Paradoxically, endocytic receptors involved in antigen uptake do not bind the majority of proteins, which may be the main reason why purified proteins stimulate at most weak immune responses. A shared feature of different types of adjuvants, capable of boosting immunogenicity of protein vaccines, is their ability to induce acute inflammation, characterized by early influx of activated neutrophils. Neutrophils are also rapidly recruited to sites of tissue injury or infection. These cells are the source of potent oxidants, including hypochlorous acid (HOCl), causing oxidation of proteins present in inflammatory foci. We demonstrate that oxidation of proteins by endogenous, neutrophils-derived HOCl increases their immunogenicity. Upon oxidation, different, randomly chosen simple proteins (yeast alcohol dehydrogenase, human and bovine serum albumin) and glycoproteins (human apo-transferrin, ovalbumin) gain the ability to bind with high affinity to several endocytic receptors on antigen presenting cells, which seems to be the major mechanism of their increased immunogenicity. The mannose receptor (CD206), scavenger receptors A (CD204) and CD36 were responsible for the uptake and presentation of HOCl-modified proteins by murine dendritic cells and macrophages. Other scavenger receptors, SREC-I and LOX-1, as well as RAGE were also able to bind HOCl-modified proteins, but they did not contribute significantly to these ligands uptake by dendritic cells because they were either not expressed or exhibited preference for more heavily oxidised proteins. Our results indicate that oxidation by neutrophils-derived HOCl may be a physiological mechanism of conferring immunogenicity on proteins which in their native forms do not bind to endocytic receptors. This mechanism might enable the immune system to detect infections caused by pathogens not recognized by pattern recognition receptors.

## Introduction

Adaptive immune responses are mainly directed against foreign (non-self) proteins. However, Th lymphocytes, which are responsible for the initiation and orchestration of immune responses, are not able to directly recognize native proteins, but require them being presented as complexes of short protein fragments (epitopes) with MHC class II molecules (MHC-II) on surfaces of antigen presenting cells (APC). Consequently, in order to induce immune responses, protein antigens have to be first taken up by APC and subjected to intracellular proteolytic processing. Two major mechanisms of antigen uptake by APC have been described: receptor-mediated endocytosis and non-specific fluid-phase macropinocytosis [1]. Nevertheless, the observation that pinocytosed antigens need to be present at even more than a thousand-times higher concentrations than antigens undergoing receptor-mediated endocytosis in order to induce an equivalent immune response [2–4], questions physiological significance of the latter mechanism in vivo. Moreover, as a result of maturation, dendritic cells (DC) which are the most proficient type of APC, shut down macropinocytosis, but they continue to capture, process, and present antigens internalised via endocytic receptors [5]. Paradoxically, although protein fragments are the only type of antigens presented in the context of both class I and II MHC molecules, the major endocytic receptors of APC, mediating antigen uptake in non-immune hosts: scavenger receptors (SR) and C-type lectins, do not bind the majority of proteins. This seems to be an important reason why purified proteins are at most weakly immunogenic. However, it is possible to boost immunogenicity of protein vaccines by co-administration of the so-called adjuvants. A shared feature of different types of adjuvants, applied in human and experimental animal vaccines, is their ability to induce acute inflammatory responses, characterized by early appearance of activated neutrophils and monocytes at the site of injection [6–8]. As a part of their microbicidal activity, these cells produce large quantities of reactive oxygen species in the process called respiratory burst. In this process, a multicomponent, membrane-anchored enzyme NADPH oxidase catalyses reduction of molecular oxygen with an electron derived from NADPH to form superoxide anion (O_2_^-^). Superoxide anion may then undergo conversion, either spontaneous or catalysed by superoxide dismutase (SOD), into hydrogen peroxide. The enzyme myeloperoxidase (MPO), being the most abundant protein of neutrophils, present in their azurophilic granules, but also expressed by monocytes utilizes H_2_O_2_ and chloride anions as substrates to synthesize a very potent oxidant—hypochlorous acid (HOCl). HOCl not only contributes significantly to microbicidal activity of neutrophils [9], but also causes indiscriminative oxidation of both host- and, if present, pathogen-derived proteins [10]. It has been demonstrated previously that, in comparison to unmodified ovalbumin (OVA), OVA treated with reagent HOCl (OVA-Cl) exhibits strongly enhanced immunogenicity [11,12]. Both increased susceptibility to digestion by proteases involved in antigen processing and enhanced uptake by APC were suggested as being responsible for increased immunogenicity of OVA-Cl. However, receptors mediating this increased uptake were not identified. It is also not known whether modification of other proteins increases their immunogenicity and whether endogenous HOCl, produced by activated neutrophils, is capable of producing a similar effect. We have addressed all these questions in the presented work. Our results indicate that modification by neutrophils-derived HOCl may be a universal, physiological mechanism of conferring immunogenicity on proteins which in their native forms do not bind to endocytic receptors involved in the uptake of antigens.

## Materials and Methods

### Native proteins and their modification with HOCl

Low endotoxin ovalbumin (OVA) was obtained from Hyglos; bovine serum albumin (BSA) from Roche Diagnostic; mouse transferrin from Rockland Immunochemicals; fatty acid and globulin free human serum albumin (HSA), human apo-transferrin (TFN), and alcohol dehydrogenase from *Saccharomyces cerevisiae* (YAD) from Sigma-Aldrich. The lipopolysaccharide (LPS) contamination of proteins was determined in a bioassay in which the ability of proteins to stimulate polymyxin B-sensitive tumour necrosis factor (TNF)-α production in macrophages was compared with effects of different concentrations of LPS. According to this assay, the LPS contamination of YAD was ~10 ng/mg protein. OVA, TFN and HSA seemed free of significant microbial contamination as they did not stimulate TNF-α production in macrophages even at 100 μg/ml. The LPS contamination of YAD was decreased to ~4 ng/mg protein by incubation with polymyxin B-agarose beads, as described previously [13].

For modification with hypochlorite, proteins were dissolved at 2 mg/ml in PBS (pH 7.4) and incubated at 37°C for 2 h with 3 mM (OVA, HSA, BSA, TFN) or 2 mM (YAD) NaOCl (Sigma-Aldrich). The corresponding HOCl:protein molar ratios were: 68:1 (OVA), 100:1 (HSA and BSA), 115:1 (TFN) and 141:1 (YAD homotetramer). Alternatively, in order to produce more or less heavy oxidation, OVA was incubated with, respectively, 6 mM (OVA-Cl_H_) or 1 mM (OVA-Cl_L_) NaOCl. The NaOCl concentration was determined before each reaction by absorbance measurements at 292 nm, using a molar extinction coefficient of 350 M^-1^ cm^-1^. To stop the reaction, the samples were treated with supra-stoichiometric amounts of thiosulphate (POCH) and then subjected to extensive, 24-h dialysis in PBS at 2–8°C. Modified proteins were aliquoted and stored at -80°C for no longer than 2 months.

Glycolaldehyde-modified BSA (GA-BSA) was prepared as described previously [13].

### Other reagents

Rat anti-mouse scavenger receptor A (SR-A) mAb (clone 2F8) was obtained from AbD Serotec; mouse anti-mouse CD36 mAb (CRF D-2712) from Hycult Biotech; mouse IgA isotype control mAb (M18-254), rat IgG2b isotype control mAb (A95-1), rat anti-mouse CD11b mAb (M1/70) and phycoerythrin (PE)-streptavidin conjugate from BD Biosciences; rat IgG2a isotype control mAb (54447), normal goat IgG, polyclonal goat anti-mouse CD36, anti-mouse LOX-1 (lectin-type oxidised LDL receptor-1), anti-human SREC-I (scavenger receptor expressed by endothelial cells-I) Ab and PE-conjugated rat anti-mouse LOX-1 mAb (214012) from R&D Systems; polyclonal goat anti-mouse SREC-I, anti-mouse RAGE (receptor for advanced glycation end products), anti-mouse stabilin-1 and rabbit anti-mouse-stabilin-1 Ab from Santa Cruz Biotechnology; PE-conjugated donkey anti-goat IgG Ab from SouthernBiotech; rat anti-mouse CD206/mannose receptor mAb (MR5D3) and PE-conjugated goat anti-rat IgG Ab from BioLegend; horseradish peroxidase (HRP)-conjugated rabbit anti-mouse IgA, F(ab’)2 fragments of goat anti-rat IgG and donkey anti-goat IgG Ab from Rockland.

Ultrapure LPS from *Escherichia coli* K12 strain was purchased from InvivoGen; dextran sulphate (DS, MW ~500 kDa), mannan from *S*. *cerevisiae* (Man) and chondroitin sulphate A sodium salt from bovine trachea (CS) from Sigma-Aldrich. Low endotoxin acetylated LDL (AcLDL) and moderately oxidised LDL (oxLDL) were obtained from Biomedical Technologies and Alexa Fluor 488-labelled AcLDL (AF-AcLDL) and DQ-OVA from Invitrogen.

Zymosan depleted with Toll-like receptor 2 (TLR2) agonists was prepared by boiling 0.5 mg/ml zymosan (Sigma-Aldrich) in 10 M NaOH for 30 min, as described previously [14]. Depleted zymosan was washed 3 times and stored at -20°C. Heat-killed *Staphylococcus aureus* bacteria (ATCC 25923) were provided by Dr. Anna Białecka (Center of Microbiological Research and Autovaccines, Cracow, Poland).

### Mice

Breeding pairs of SR-A-deficient, CD36-deficient, MPO-deficient and OT-II transgenic mice, all on the C57BL/6 background, as well as wild-type C57BL/6 (WT) and CBA mice were purchased from the Jackson Laboratory. The mannose receptor (MR)-deficient mice were obtained by cross-breeding of heterozygotic MR+/- mice (the Jackson Laboratory). MR-/- mice were identified in the progeny of MR+/- mice by PCR genotyping, using the protocol and primers recommended by the Jackson Laboratory. Mice were housed in our facility in microisolator cages with filter tops on a 12-h light/dark cycle. This study was carried out in strict accordance with the recommendations in the Guide for the Care and Use of Laboratory Animals of the Ministry of Science and Informatization of Poland. The protocol was approved by the I Local Committee on the Ethics of Animal Experiments of Jagiellonian University (Permit Number: 83/2009). All surgery was performed under isoflurane anaesthesia, and all efforts were made to minimize suffering.

### Cells

Twelve-sixteen weeks old male mice were quickly euthanized by overdosing of isoflurane vapours (Abbott Laboratories) followed by cervical dislocation. Inflammatory peritoneal cells, elicited with 1.5 ml of 3% Thioglycollate (Difco Laboratories), injected *i*.*p*. 4 days earlier, were washed out with PBS. The cells were re-suspended in FCS-RPMI [RPMI 1640 medium with 25 mM HEPES (Lonza), supplemented with 10% foetal calf serum (FCS, Cytogen), 2 mM stable L-glutamine (Cytogen), and 0.04 mg/ml gentamycin (KRKA)] and plated in 96- or 24-well tissue culture treated plates. After overnight incubation, non-adherent cells were removed by washing and adherent macrophages (PEM) were used in experiments described below.

Dendritic cells were differentiated from bone marrow cells of 4–6 weeks old female mice under the influence of 20 ng/ml rGM-CSF (eBioscience), as described previously [15]. CD11c-positive DC (BM-DC) were purified from 6-days cultures with the use of magnetic beads (Miltenyi Biotec). In experiments with BM-DC IMDM medium (Lonza) containing 10% FCS, glutamine and gentamycin (FCS-IMDM) was used.

CHO-K1 cells stably transfected with human CD36 (CRL-2092) and non-transfected CHO-K1 cells (CCL-61) were obtained from ATCC and cultured in F12 medium (ATCC) supplemented with 10% FCS. The J774A.1 macrophage-like cell line (TIB-67) was cultured in FCS-RPMI.

In order to prepare single cell suspension, spleens were cut into smaller pieces and minced between frosted parts of microscopic slides. Tissue debris were separated by passing through a 70 μm nylon cell strainer (BD Falcon) and the cells were washed twice with PBS. CD4-positive cells were isolated with the use of magnetic beads (Miltenyi Biotec), according to the manufacturer’s instruction.

### Binding experiments with isolated receptors

#### Preparation of coated plates and the shared procedures

Unless otherwise stated, 96-well half-area ELISA plates (Corning Incorporated) were coated overnight at 4–8°C with 50 μl/well of 20 μg/ml solutions of different proteins in PBS. The next day the plates were washed twice with 0.05% Tween 20 in PBS (Tween/PBS). All subsequent steps were performed at room temperature. Between addition of consecutive reagents plates were washed 4 times with Tween/PBS. All recombinant receptors were obtained from R&D Systems. The enzymatic reaction was performed with the use of TMB Substrate Reagent Set (BD Biosciences) as the HRP substrate.

#### Recombinant mouse LOX-1 (rLOX-1)

Wells were blocked for 1 h with 0.12 ml of 1% BSA and 5% sucrose in PBS. His-tagged rLOX-1 at 1 μg/ml in 50 μl of PBS supplemented with 0.1% Tween 20 and 1% BSA was allowed to bind to coated plates for 70 min. rLOX-1 bound to plates was quantified with 0.9 μg/ml of goat anti-mouse LOX-1 Ab (1 h), followed by 3 μg/ml of HRP-conjugated donkey anti-goat IgG Ab (1 h) in 1% BSA.

#### Recombinant mouse MR (rMR)

Plates were blocked with 10% FCS in PBS. The wells were subsequently filled with 50 μl of 2 μg/ml His-tagged rMR in 10% FCS in PBS with Ca and Mg (Lonza) for 70-min incubation. Bound rMR was detected with either 1:400 dilution of HRP-conjugated mouse monoclonal IgG1 anti-6×Histidine (R&D Systems, competition experiments with Ab) or with 2.5 μg/ml of rat anti-mouse CD206 mAb (1 h), followed by 0.5 μg/ml of HRP-conjugated goat anti-rat IgG Ab (1 h) in 10% FCS.

#### Recombinant human SREC-I (rSREC-I)

Plates were blocked with 10% BSA. rSREC-I/SCARF1 Fc chimera at 1 or 1.5 μg/ml in 10% BSA was added to plates for 1 h. Bound SREC-I was detected with 1.5 μg/ml of goat anti-human SREC-I Ab (1 h), followed by 4 μg/ml of HRP-conjugated anti-goat IgG Ab (1 h) in 2% BSA.

#### Recombinant mouse RAGE (rRAGE)

Plates were blocked with 10% FCS. rRAGE Fc chimera at 2 μg/ml in 10% FCS was allowed to bind to coated plates for 70 min. Bound rRAGE was detected with 4 μg/ml of goat anti-mouse RAGE Ab (1 h), followed by 3 μg/ml of HRP-conjugated anti-goat IgG Ab (1 h) in 10% FCS.

#### Recombinant mouse CD36 (rCD36)

Plates were blocked with 10% FCS. rCD36/SR-B3 Fc chimera at 2 μg/ml in 10% FCS was allowed to bind to coated plates for 70 min. Bound rCD36 was detected with 1 μg/ml of goat anti-mouse CD36 Ab (1 h), followed by 2 μg/ml of HRP-conjugated anti-goat IgG Ab (1 h) in 10% FCS.

#### Competition experiments

Soluble ligands were pre-incubated for 30 min at room temperature with recombinant receptors before the mixture was added to coated plates. The rest of procedures was the same as described above.

#### Binding of natural SR-A

Binding of SR-A, present in a lysate of C57BL/6 PEM, to protein-coated plates was performed, as described previously by others [16]. Lysate prepared from SR-A-deficient PEM served as the control for non-specific binding.

### Assessment of surface protein expression

#### Flow cytometry

PEM (detached by 15 min incubation at 37°C with 15 mM lidocaine (Sigma-Aldrich) plus 5 mM EDTA in PBS), purified BM-DC or unfractionated splenic cells (2 × 10^5^ leukocytes) were pre-incubated for 30 min on ice in 0.1 ml of FCS-RPMI containing 40% mouse serum and 50 μg/ml of non-immune mouse IgG2a (BD Biosciences), in order to decrease non-specific binding. Subsequently, 0.1 ml solutions of receptor-specific or control mAb were added, to give final mAb concentrations of 10 μg/ml, and the incubation was continued for 50 min. Unbound mAb were removed by washing with 2 ml of ice-cold PBS, and the cells were incubated for another 50 min with 5 μg/ml of PE-conjugated secondary Ab in 0.2 ml FCS-RPMI. In order to enable identification of DC and macrophages, splenic cells were subjected to additional incubation with 5 μg/ml of allophycocyanin-conjugated anti-CD11c (clone N418) or F4/80 mAb (eBioscience), respectively. Following washing twice, binding of fluorescently-labelled Ab to cells was assessed by flow cytometry.

The surface expression of MHC-II, CD40 and CD86 was also determined by flow cytometry, by direct labelling of these molecules with 5 μg/ml of PE-conjugated MHC-II-, CD40-, or CD86-specific or isotype-matched control mAb obtained from BD Biosciences or eBioscience.

#### Cellular ELISA

Expression of CD36 on the surface of adherent CHO cells was determined by cellular ELISA, as described previously [17]. Primary mAb were used at 10 μg/ml and HRP-conjugated rabbit anti-mouse IgA Ab at 5 μg/ml.

### Uptake of fluorescently-labelled ligands

Native and HOCl-modified proteins were conjugated with amine-reactive, fluorescent dyes: Alexa Fluor 647 carboxylic acid or pHrodo Red succinimidyl esters, according to manufacturer’s instructions (Invitrogen). Unbound dyes were separated by extensive dialysis. The degree of labelling was estimated to be ~2:1 in the case of OVA, ~3:1 in the case of HSA and ~7.5:1 in the case of YAD. Degrees of labelling of HOCl-modified proteins were slightly (4–14%) lower than those of their native counterparts.

BM-DC (1.5 × 10^5^) were distributed into 5 ml polypropylene round-bottom tubes (BD Falcon) and pre-incubated for 20 min at room temperature with double-concentrated solutions of blocking ligands in 0.2 ml FCS-IMDM: 0.2 mg/ml AcLDL, oxLDL, DS or CS, 6 mg/ml mannan, 1 mg/ml native or HOCl-modified unlabelled proteins, 40 μg/ml receptor-specific or isotype-matched control Ab. Subsequently, 0.2 ml of solutions of fluorescently-labelled proteins were added (the final concentration 5 μg/ml) and the incubation was continued for 1 h (Alexa Fluor 647-labelled ligands) or 2 h (pHrodo-labelled ligands) in a cell culture incubator. Unbound ligands were removed by washing twice with 1 ml PBS, and the cell-associated fluorescence was quantified by flow cytometry. In the case of pHrodo-labelled ligands, for the measurement the cells were suspended in PBS which pH was adjusted to 9.0 with 25 mM HEPES, in order to allow selective quantification of ligands internalised into acidic intracellular compartments. Results of experiments in which uptake of different proteins was compared were normalized for differences in degrees of labelling, by dividing by factors equal degree of labelling of a given protein/degree of labelling of OVA-Cl.

In the case of PEM and CHO cells the uptake experiments were performed on adherent cells. Peritoneal exudate cells and CHO cells were plated in 24-well plates at 4 and 2 × 10^5^/well, respectively, and allowed to adhere overnight in 1 ml FCS-RPMI. Adherent cells were incubated with fluorescently-labelled ligands in 0.6 ml FCS-RPMI, as described above, washed once with PBS, detached with lidocaine/EDTA and their fluorescence was measured by flow cytometry.

Antigen degradation was assessed with the use of DQ-OVA (Invitrogen). DQ-OVA is a preparation of OVA which is densely substituted with the BODIPY fluorescent dye. Its fluorescence strongly increases upon proteolytic degradation due to de-quenching. BM-DC were pre-incubated or not with DS or mannan and then allowed to bind 20 μg/ml DQ-OVA during 1-h incubation on ice. Unbound DQ-OVA was removed by washing twice with ice-cold PBS and the cells were transferred to a cell culture incubator for 2-h incubation. Fluorescence of cells (FL1) was measured by flow cytometry.

For assessing effects of receptor ligation on intracellular degradation of antigens, peritoneal exudate cells and BM-DC were plated at 1.6 and 1.2 × 10^5^/well, respectively, in 96-well Optilux Black/Clear Bottom plates (BD Biosciences). The next day, adherent cells were incubated for 70 min on ice with 20 μg/ml of DQ-OVA or pHrodo-labelled OVA-Cl (pHr-OVA-Cl). Unbound ligands were removed by washing twice, and the cells were incubated for 15 min at 37°C in medium alone, in order to enable internalisation of ligands, and then for additional 2 h in medium containing 0.1 mg/ml DS, 2 mg/ml mannan, 20 μg/ml anti-SR-A 2F8 mAb or 200 ng/ml LPS. Following washing once, cell-associated fluorescence was measured with the use of a fluorescence plate reader (Infinite M200 PRO, Tecan).

### Antigen presentation in vitro

Peritoneal exudate cells were plated at 1 × 10^5^/well of 96-well plates in 0.2 ml FCS-RPMI. BM-DC were plated at 0.75 × 10^5^/well and incubated overnight in 0.15 ml of FCS-IMDM containing 20 ng/ml GM-CSF. Adherent PEM or BM-DC were incubated for 3.5 h at 37°C with 7 μg/ml OVA-Cl or 20 μg/ml OVA in 0.2 ml of FCS-RPMI or FCS-IMDM, respectively. When indicated, 200 ng/ml of LPS was added for the last 2 h of incubation with antigens. The cells were washed once with 0.18 ml of fresh medium, and 1 × 10^5^ of CD4^+^ cells/well, isolated from spleens of OT-II mice, were added in 0.2 ml of FCS-IMDM, supplemented with 0.05 mM 2-mercaptoethanol and 1 mM sodium pyruvate (Gibco) (the complete FCS-IMDM medium). Alternatively, freshly-isolated BM-DC were pulsed for 2 h with different concentrations of OVA or OVA-Cl in polypropylene tubes, before being washed and used as APC in the co-culture with CD4^+^ OT-II splenocytes.

Following 1- or 2-days co-incubation, aliquots of culture supernatants were collected for cytokine determinations by standard sandwich ELISA [18]. In some cases, lymphocyte proliferation was assessed by adding 1 μCi/well of ^3^H-thymidine (PerkinElmer) for the last 20 h of culture and quantifying radioactivity incorporated by lymphocytes with the use of a microplate scintillation counter (MicroBeta TriLux, Wallac). Before the measurement, the cells were harvested onto a glass fiber filtermat (Wallac) and after drying the filters were melted in a solid scintillator (Meltilex, PerkinElmer).

In the experiments aimed at elucidation of mechanisms of antigen presentation, 2.5 × 10^5^ of purified BM-DC were co-incubated with 7.5 × 10^5^ of CD4-positive OT-II splenocytes and 10 μg/ml OVA or OVA-Cl for 2 days in 1 ml of complete FCS-IMDM medium in 24-well plates. To block MHC-II, DC were pre-incubated for 30 min at room temperature with functional grade-purified, biotinylated rat anti-mouse MHC-II mAb (clone M5/114.15.2, eBioscience), before being added to the co-culture (final mAb concentration 5 μg/ml). To block CD40L, Th lymphocytes were pre-incubated for 30 min with no azide/low endotoxin hamster anti-mouse CD154 mAb (clone MR1, BD Biosciences; 25 μg/ml). Cytokine concentrations in culture supernatants were determination by ELISA. Adherent BM-DC were detached with lidocaine/EDTA, combined with non-adherent cells, and expression of MHC-II, CD40 and CD86 on BM-DC, distinguished thanks to co-staining with allophycocyanin-conjugated anti-CD11c mAb (HL3, BD Biosciences), was determined as described above, except that biotinylated anti-MHC-II followed by PE-conjugated streptavidin, instead of directly PE-labelled anti-MHC-II mAb, was used to determine the expression level of MHC-II.

### Presentation of neutrophils-modified OVA

#### Assessing production of reactive oxygen species

Lucigenin-enhanced chemiluminescence is known to be caused by extracellular superoxide anion [13]. In contrast, luminol-enhanced chemiluminescence is not as selective but it depends on HOCl formation [19]. Peritoneal exudate cells, elicited with 1.5 ml of 3% Thioglycollate, injected ***i*.*p*.** 18 h earlier and consisting mainly of neutrophils [20], were washed out with PBS and re-suspended in Ca/ Mg- and glucose-containing Hanks' balanced salt solution (HBSS, Lonza) at 1 × 10^7^/ml. Wells of 96-well plates (F16 Black MaxiSorp, Nunc) were filled with 100 μl of cell suspension, 50 μl of luminol (the final concentration 0.5 mg/ml) or lucigenin (0.2 mg/ml) solution in HBSS (Sigma-Aldrich) and 50 μl HBSS with or without 5 kU/ml bovine SOD (Calbiochem) or 0.5 mM 4-aminonbenzoic acid hydrazide (ABAH, Sigma-Aldrich). After 25 min incubation at 37°C in a cell culture incubator, 50 μl of zymosan (0.2 mg/ml) or heat-killed ***S*. *aureus*** (75 × 10^6^/ml) was added and then immediately chemiluminescence was recorded for 75 min in a temperature-stabilised (37°C) luminometer (Lucy 1, Anthos).

#### Presentation of neutrophils-modified OVA by PEM in vitro

Peritoneal exudate cells were plated at 1.8 × 10^5^/well in 96-well plates. The next day, PEM were co-incubated for 2 h 15 min in a cell culture incubator with 2.5 × 10^6^/ml neutrophils, 75 × 10^6^/ml heat-killed ***S*. *aureus*** and 25 μg/ml OVA in 0.2 ml of HBSS. When indicated, 5 kU/ml of SOD was additionally included in the incubation mixture. Fifty μl of 50 mM EDTA was added for the last 15 min of incubation to facilitate detachment of neutrophils, and the majority of neutrophils, together with free bacteria and OVA, were removed by extensive washing. PEM were then incubated for 24 h with 2 × 10^5^/well OT-II Th lymphocytes in 0.2 ml of complete FCS-IMDM medium.

### In vivo antibody production

WT and MPO-/- mice were immunized by *i*.*p*. injection with 1 ml of PBS with Ca/Mg, containing 20 μg HSA, OVA or YAD and an adjuvant. As adjuvants: 0.2 mg of depleted zymosan, 2 × 10^8^ heat-killed *S*. *aureus*, 2 μg Pam3CSK lipopeptide (InvivoGen), 2 μg LPS or 50 ng recombinant mouse TNF-α (eBioscience) plus 1.7 μg WKYMVm peptide (a synthetic agonist of receptors for chemotactic formylated peptides, Tocris) per ml per mouse were used. WT, SR-A-/- and CD36-/- mice were immunized with 20 μg and CBA mice with 10 or 100 μg of proteins in 1 ml PBS. Fourteen days later some mice received second *i*.*p*. injection with 20 μg of native proteins alone in 0.5 ml PBS. Blood was collected 8 days after first (primary response) or second (secondary response) immunization.

Titers of specific immunoglobulins in sera were determined as follows. Half-area ELISA plates were coated with 50 μl/well of 10 μg/ml proteins in PBS by overnight incubation at 4–8°C. The next day the plates were washed twice with Tween/PBS and blocked for 1 h with 10% FCS. Eight 3-fold serial dilutions of sera were prepared, starting from 1:5 or 1:10, and added to plates for 1-h incubation at room temperature. One % BSA in PBS served as the reagent diluent. Following washing, the wells were filled for 45 min with 26 μl of biotin conjugated isotype-specific Ab: 25 ng/ml goat anti-mouse IgG Ab, 50 ng/ml goat anti-mouse IgG2a Ab, 100 ng/ml goat anti-mouse IgM Ab (SouthernBiotech) or 1:20,000 dilution of rat anti-mouse IgG1 Ab (ICN Biomedicals). Subsequently, 50 μl of 1 μg/ml (total IgG, IgG1 and IgM assays) or 0.5 μg/ml (the IgG2a assay) streptavidin-HRP conjugate (Vector Laboratories) was added for 45 min. The plates were subjected to the final washing (4 times) and the enzymatic reaction for peroxidase was performed with the use of 3.5 mM H_2_O_2_ and 0.5 mg/ml o-phenylenediamine dihydrochloride (Sigma-Aldrich) in 0.1 M phosphate-citrate buffer (pH 5.0) as substrates. Thirty min later the reaction was stopped with 3M H_2_SO_4_ and absorbance at 492 nm of the product was measured with the use of a plate reader (PowerWave, Bio-Tek Instruments). In parallel, non-specific binding to wells not coated with antigens was determined for each dilution of serum and subtracted from the total binding. This calculated specific binding was plotted as the function of logarithm of serum dilution. The titer was defined as the dilution of serum which binds specifically with the absorbance value of 0.2 and was determined by fitting a sigmoidal dose-response non-linear regression curve with the use of GraphPad Prism software.

### Data analysis

After confirming homogeneity of variances with the F test, experimental groups were compared with control groups with the Student’s t-test (GraphPad Prism software) and the p values < 0.05 were assumed to denote statistically significant difference. One way ANOVA was applied for multiple comparisons, with the Tukey-Kramer post-test used to compare all pairs of groups, the Bonferroni test used to compare selected groups, and the Dunnett’s test to compare all other groups to the control group.

## Results

### Increased immunogenicity of OVA-Cl may be caused by its enhanced uptake by APC

**Fig 1:**
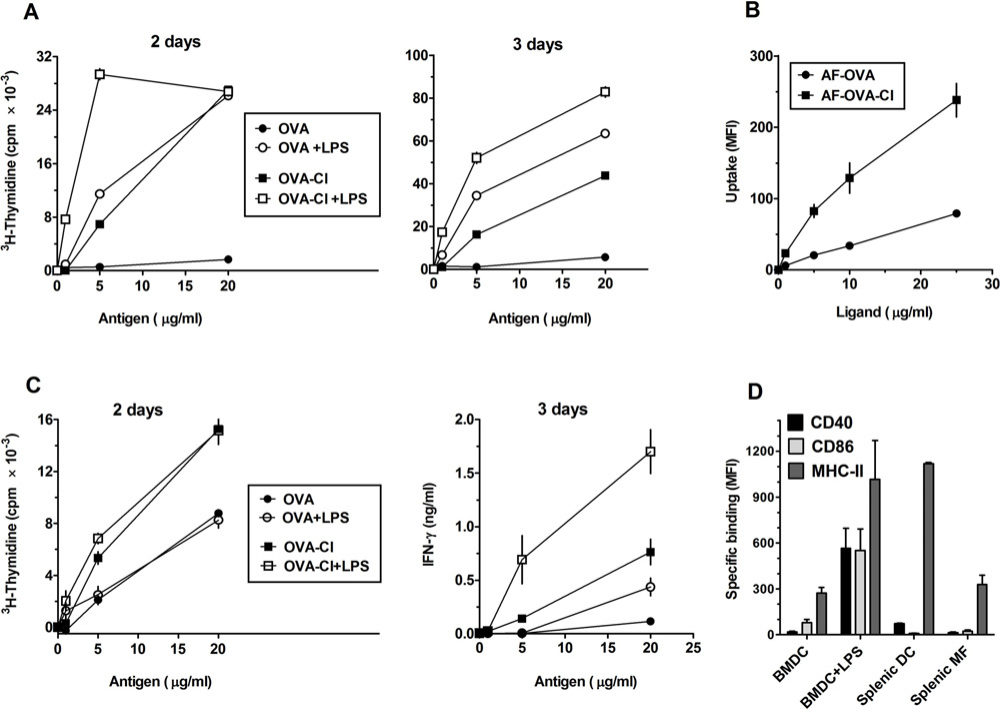
OVA-Cl exhibits increased immunogenicity that may be caused by enhanced uptake by APC. (**A**) BM-DC were incubated with indicated concentrations of OVA or OVA-Cl for 2 h at 37°C. When indicated, 200 ng/ml LPS was additionally included. Following washing, 0.4 × 10^5^ BM-DC were co-cultured for 2 or 3 days with 1.5 × 10^5^ CD4^+^ OT-II lymphocytes. One μCi of ^3^H-thymidine was added for the last 20 h of co-culture and radioactivity incorporated by proliferating lymphocytes was measured by scintillation counting. (**B**) BM-DC were incubated for 1 h at 37°C with indicated concentrations of Alexa Fluor 647-labelled OVA (AF-OVA) or OVA-Cl (AF-OVA-Cl) and, following washing, cell-associated fluorescence was measured by flow cytometry. (**C**) Unfractionated splenocytes, prepared from spleens of OT-II mice, we pre-incubated with OVA, OVA-Cl and LPS, as described in A, washed, plated at 2.5 × 10^5^/well in 0.2 ml of fresh medium and cultured for 2 days, for assessing lymphocyte proliferation, or 3 days, for assessing IFN-γ level in culture medium by ELISA. Results shown on graphs A-C are averages ± SEM of 2 (B), 3 (A) or 4 (C) replicates, obtained in single experiments which were repeated at least 3 times with similar results. (**D**) Expression of MHC-II and co-stimulatory molecules on the surface of BM-DC as well as splenic DC and macrophages was determined by flow cytometry. When indicated, BM-DC were pre-incubated overnight with LPS. Specific binding was calculated by subtracting binding of PE-conjugated control mAb from the total binding of specific mAb. The results shown are averages ±SEM from 4–6 independent experiments.

Previously, OVA-Cl has been shown to be presented effectively by BM-DC to memory Th lymphocytes [12], known to be more easily activated than naïve lymphocytes. We have found that BM-DC, pre-incubated for 2 h with OVA-Cl, are also able to stimulate proliferation of naïve Th lymphocytes (Fig 1A). In the presence of LPS, lymphocyte proliferation during 2 days of co-culture was stimulated by lower concentrations of OVA-Cl than OVA (Fig 1A, *left panel*). During 3-days co-culture the difference in the sensitivity of detection between OVA and OVA-Cl disappeared, but OVA-Cl stimulated more intense proliferation of CD4^+^ lymphocytes than OVA in the entire range of concentrations tested (Fig 1A, *right panel*). In contrast, in the absence of LPS, OVA-Cl was effectively presented to lymphocytes at a concentration as low as 5 μg/ml, whereas OVA did not stimulate lymphocyte proliferation even at 20 μg/ml (Fig 1A).

It is believed that except of specific recognition of antigen-MHC complexes on the surface of DC, activation of naïve lymphocytes during antigen presentation requires additional stimuli, provided by co-stimulatory molecules on the surface of DC (second signal) and by DC-derived cytokines (third signal). However, we found that, in contrast to LPS, neither OVA nor OVA-Cl at concentrations up to 50 μg/ml stimulated cytokine release from BM-DC or affected expression of MHC-II or co-stimulatory molecules (CD40 and CD86) on their surface (S1 Fig).

**Fig 2:**
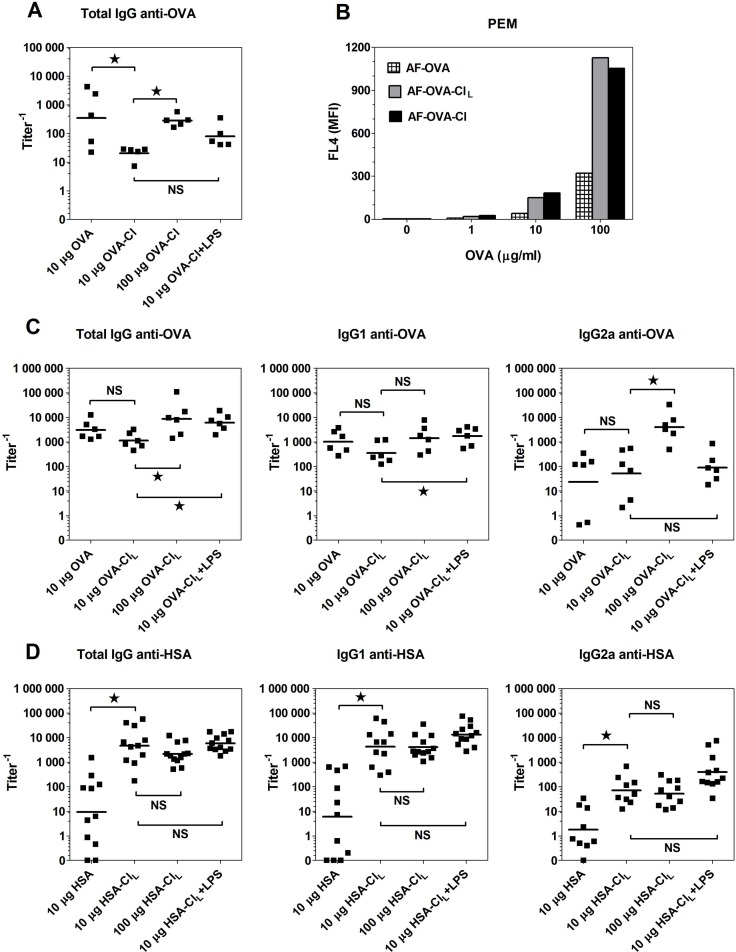
Humoral immune response stimulated by native and HOCl-oxidised proteins in vivo. (**A**, **C**, **D**) CBA mice were immunized with the indicated antigens. Two weeks later all mice received boost immunization with 20 μg of native proteins and 8 days later sera were collected for the determination of titers of specific Ab by ELISA. Points represent titer values in individual mice and horizontal lines geometric means. (**B**) Uptake of fluorescently-labelled ligands by PEM was determined by flow cytometry. The data were analysed by ANOVA and the Benferroni post-test was applied to compare the indicated pairs of columns. *, p < 0.05; NS, non-significant.

Another potential cause of increased immunogenicity of OVA-Cl may be enhanced uptake and intracellular processing by APC. In order to test this possibility, we labelled OVA and OVA-Cl with the Alexa Fluor 647 fluorescent dye (AF) and studied their uptake by APC. We found that both PEM (Fig 2B) and BM-DC (Fig 1B) endocytosed much more AF-OVA-Cl than AF-OVA. Of note, stimulation of lymphocyte proliferation in the presence of LPS correlated with the level of antigen uptake (compare Fig 1A, *left panel*, and 1B). Thus, it seems that in the presence of LPS the major limiting factors in antigen presentation are amounts of endocytosed antigens and the ability of BM-DC to process and present these antigens. Consistent with this interpretation, increasing the duration of co-culture to 3 days resulted in the shift of OVA-Cl concentrations producing the maximal effect to higher concentrations (Fig 1A, *right panel*). In contrast, there was no correlation between antigen uptake and lymphocyte proliferation in the absence of LPS (Fig 1A and 1B), which indicates that also factors other than enhanced uptake contribute to increased immunogenicity of HOCl-modified OVA.

### Stimulation of pattern recognition receptors on APC is not required for triggering of primary immune response to OVA-Cl

**Fig 3:**
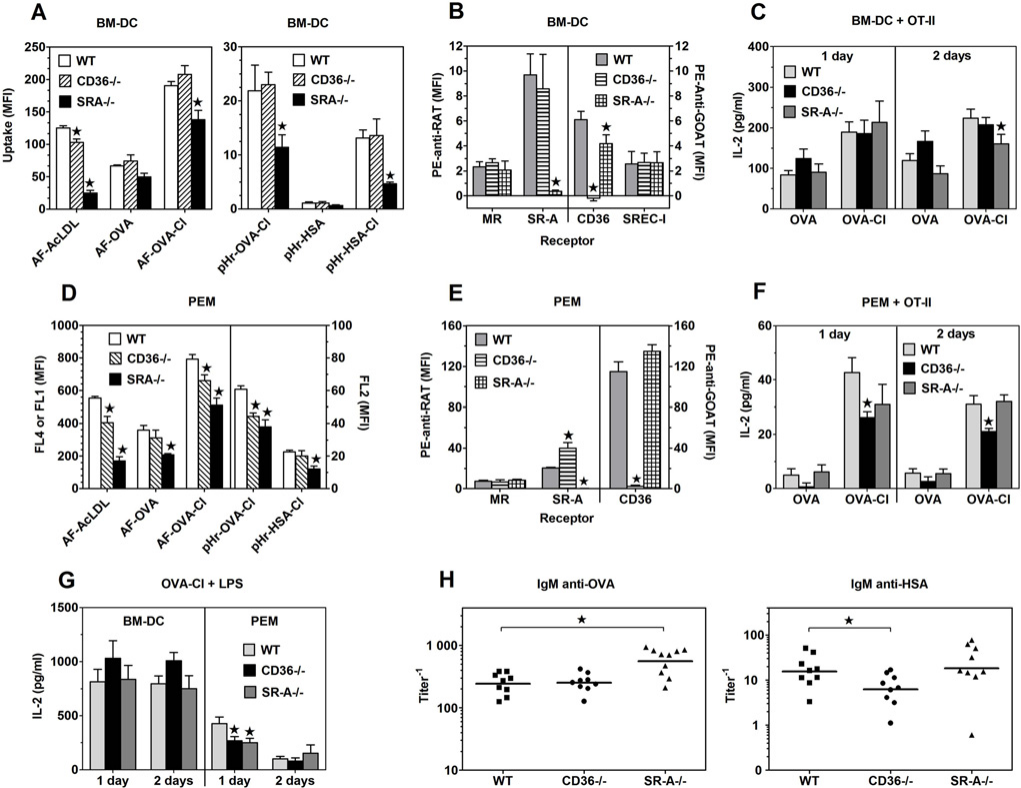
Effects of SR-A or CD36 deficiency on antigen uptake, expression of endocytic receptors and antigen presentation to CD4^+^ OT-II splenocytes. (**A**, **D**) Uptake of indicated, fluorescently labelled proteins, present at 5 μg/ml by BM-DC (A) and PEM (D) was assessed by flow cytometry. (**B**, **E**) Specific binding of Ab to receptors (geometric mean fluorescence intensity) on BM-DC (B) and PEM (E) which was obtained by subtracting binding of control Ab from the total binding of receptor-specific Ab. (**C**, **F**) IL-2 production in 1- or 2-days co-cultures of BM-DC (C) or PEM (F) with CD4^+^ OT-II lymphocytes. Directly before the co-incubation with lymphocytes, APC were pulsed for 3.5 h with 20 μg/ml OVA or 7 μg/ml OVA-Cl. The data shown on graphs A-G are means +SEM from 6–8 independent experiments. (**H**) Titers of OVA- or HSA-specific IgM in sera of mice immunized 8 days earlier with 20 μg OVA-Cl or HSA-Cl. Points represent titer values in individual mice and horizontal lines geometric means. The data were analysed with the regular (H) or repeated measures (A-G) ANOVA and the Dunnett’s post-test was used to make comparisons with the control groups (WT). *, p < 0.05.

Efficient presentation to naïve CD4^+^ lymphocytes by DC of protein antigens alone, not contaminated with microbial products, is inconsistent with the currently dominating paradigm, according to which such presentation requires activation of pattern-recognition receptors (PRR) on DC [21]. As DC activation might be caused by GM-CSF used in the culture of BM-DC, we decided to assess whether OVA-Cl alone would also be able to initiate immune response in the culture of splenocytes isolated from naïve OT-II mice. Also in the culture of unfractionated splenocytes OVA-Cl stimulated proliferation of lymphocytes which was higher than that stimulated by OVA (Fig 1C, *left panel*). There were, however, two major differences with previous experiments employing BM-DC. First, in the case of unfractionated splenocytes proliferation was also stimulated by native OVA. This difference might be caused by higher expression of endocytic receptors on splenic DC than on BM-DC or the presence among splenocytes of other cell types. Consistent with the former possibility, also BM-DC effectively presented OVA when they were allowed to endocytose it for a longer period (Fig 3C). Second, unlike in the co-culture with BM-DC, in unfractionated splenocytes lymphocyte proliferation was not enhanced by LPS. However, under these conditions LPS did promote differentiation of naïve T lymphocytes towards Th1 cells, as indicated by strongly increased interferon-γ (IFN-γ) production. Interestingly, very high IFN-γ production was also stimulated by OVA-Cl alone, which was not only higher than that stimulated by OVA alone but also by OVA plus LPS (Fig 1C, *right panel*).

Mechanisms of immunostimulation exerted by PRR agonists are thought to involve up-regulation of MHC and co-stimulatory molecules expression on DC, known as maturation. Therefore, attempting to identify factors responsible for differences in APC functions between splenic APC and BM-DC, we examined expression of these molecules on both cell types. We found that splenic DC, defined in flow cytometry as large cells with high expression level of CD11c, expressed as much as ~4-times more of both MHC-II and CD40 than BM-DC. Expression of these molecules on BM-DC was similar to that on splenic F4/80-positive macrophages (Fig 1D). Treatment with LPS induced strong expression of CD40 and CD86 as well as up-regulated expression of MHC-II on BM-DC to the level seen on splenic DC. Thus, BM-DC, but not splenic DC, may require up-regulation of MHC-II expression for optimal antigen presentation, which might explain why LPS enhances proliferation of lymphocytes in the co-culture with BM-DC, but not in the suspension of unfractionated splenocytes.

**Fig 4:**
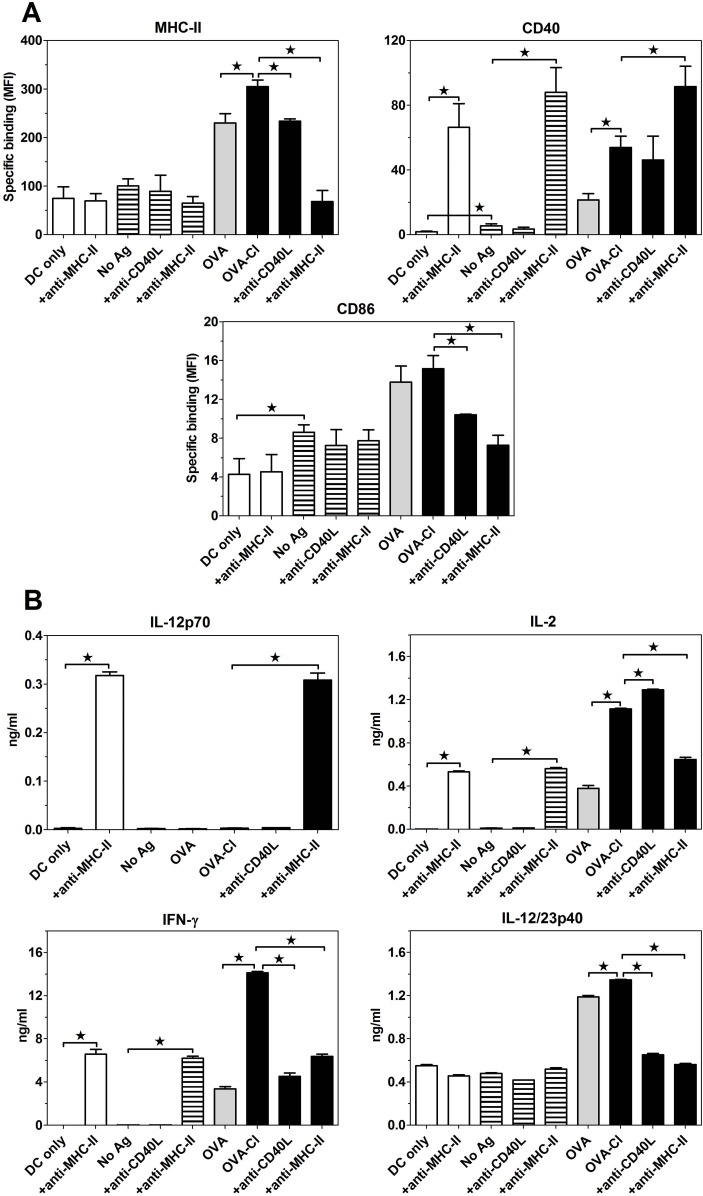
Expression of MHC-II and co-stimulatory molecules on BM-DC (A) and cytokine production (B) in the co-culture of BM-DC with OT-II Th lymphocytes. Purified BM-DC (2.5 × 10^5^) were co-cultured with CD4^+^ OT-II splenocytes (7.5 × 10^5^) for 2 days in 1 ml of medium, with or without 10 μg/ml OVA or OVA-Cl. When indicated, 5 μg/ml of blocking anti-MHC-II mAb or 25 μg/ml anti-CD40L mAb was additionally included. Expression of proteins on BM-DC surfaces was assessed by flow cytometry and cytokine concentration in culture supernatants by ELISA. The results shown are averages +SEM from 4 independent experiments (A) or means +SEM of 4 replicates obtained in a single, representative experiment (B). The data were analysed by ANOVA, combined with the Tukey-Kramer post-test. *, p < 0.05.

In order to gain insight into mechanisms of this efficient Ag presentation in the absence of PRR ligands, we assessed effects of co-culturing BM-DC and OT-II CD4^+^ T cells, with or without OVA antigens, on the expression of MHC-II and co-stimulatory molecules on BM-DC as well as on cytokine release. Even in the absence of specific antigens, 2-days co-culture of BM-DC with CD4^+^ T lymphocytes resulted in slight up-regulation of CD40 and CD86 expression on BM-DC (Fig 4A). BM-DC also spontaneously released interleukin (IL)-12p40 subunit, but no production of IL-2 and IFN-γ was observed in the absence of antigen (Fig 4B). Inclusion of OVA in the co-culture led to strong up-regulation of MHC-II and co-stimulatory molecules expression on BM-DC, enhanced IL-12p40 and induced IL-2 and IFN-γ secretion. In comparison to OVA, OVA-Cl induced much higher CD40 expression as well as IL-2 and IFN-γ secretion, but similar MHC-II and CD86 expression. Despite stimulating much higher IFN-γ production, OVA-Cl stimulated similar IL-12p40 production as OVA. Moreover, no detectable production of bioactive IL-12p70 was observed in the co-culture, which, together, indicate that in this system IFN-γ production is IL-12-independent. The role of recognition of peptide-MHC-II complexes on BM-DC by Th lymphocytes as well as of CD40-CD40L interactions in the observed phenomena was assessed with the use of blocking mAb. Blocking MHC-II reversed completely OVA-Cl-stimulated increases of MHC-II and CD86 expression (Fig 4A) as well as of IL-2, IFN-γ and IL-12p40 secretion (Fig 4B). Surprisingly, while anti-MHC-II mAb blocked recognition of peptide-MHC-II complexes by Th lymphocytes, binding of this mAb to BM-DC gave rise to selective activation of these cells, reflected by very strong up-regulation of CD40, but not MHC-II or CD86 expression, and IL-2 production by BM-DC themselves. Acting on BM-DC anti-MHC-II mAb also induced production of IL-12p70, likely related to the induction of IFN-γ production in these cells. In turn, blocking interactions between CD40 and CD40L with the use of anti-CD40L mAb, largely reversed the OVA-Cl-stimulated up-regulation of CD86, but not CD40 expression, and OVA-Cl-stimulated IL-12p40 and IFN-γ secretion, but had no such effect on IL-2 production.

For assessing the type of immune responses stimulated by OVA-Cl in vivo, we have chosen the CBA strain of mice because the IgG2a isotype of Ab is not produced by the C57BL/6 mouse strain [22]. However, immunization with native OVA induced already very high production of OVA-specific Ab, which was higher than that induced by OVA-Cl (Fig 2A). These results confirm what was already suggested in a previous report [12] that, unlike the epitope recognized by the transgenic TCR of OT-II lymphocytes, some other epitopes present in OVA are destroyed by HOCl-mediated oxidation. Consistent with this interpretation, immunization of mice with less heavily oxidised OVA-Cl_L_ (which was treated with 1 mM instead of 3 mM HOCl) induced much higher (~56-times) titers of OVA-specific Ab than OVA-Cl (Fig 2C), despite the fact that the uptake of AF-OVA-Cl_L_ by APC was indistinguishable from that of AF-OVA-Cl (Fig 2B). In contrast, HSA, which uptake by APC was much lower than that of OVA (Fig 3A), stimulated very low production of specific Ab, but upon oxidation its immunogenicity dramatically increased, as indicated by on average more than 500-times higher titer of HSA-specific IgG. At this low dose (10 μg) both native and HOCl-oxidised proteins stimulated Th2-polarised humoral responses, as indicated by much higher production of specific IgG of the IgG1 than the IgG2a isotype (Fig 2C and 2D). Increasing the dose of OVA-Cl_L_ during priming, from 10 to 100 μg, not only increased the magnitude of humoral response (~8-times), but also caused its Th1-polarization, as titers of the IgG1 isotype increased only ~4-times, whereas those of the IgG2a isotype as much as ~80-times (Fig 2C). In contrast, increasing the dose of HSA-Cl_L_ affected neither the magnitude nor the polarization of humoral response (Fig 2D). Surprisingly, the use of LPS as the adjuvant did not reverse the Th2 polarisation of HSA-Cl_L_- or OVA-Cl_L_-stimulated immune response.

### Increased binding to DC is a shared feature of different chlorinated proteins and glycoproteins

**Fig 5:**
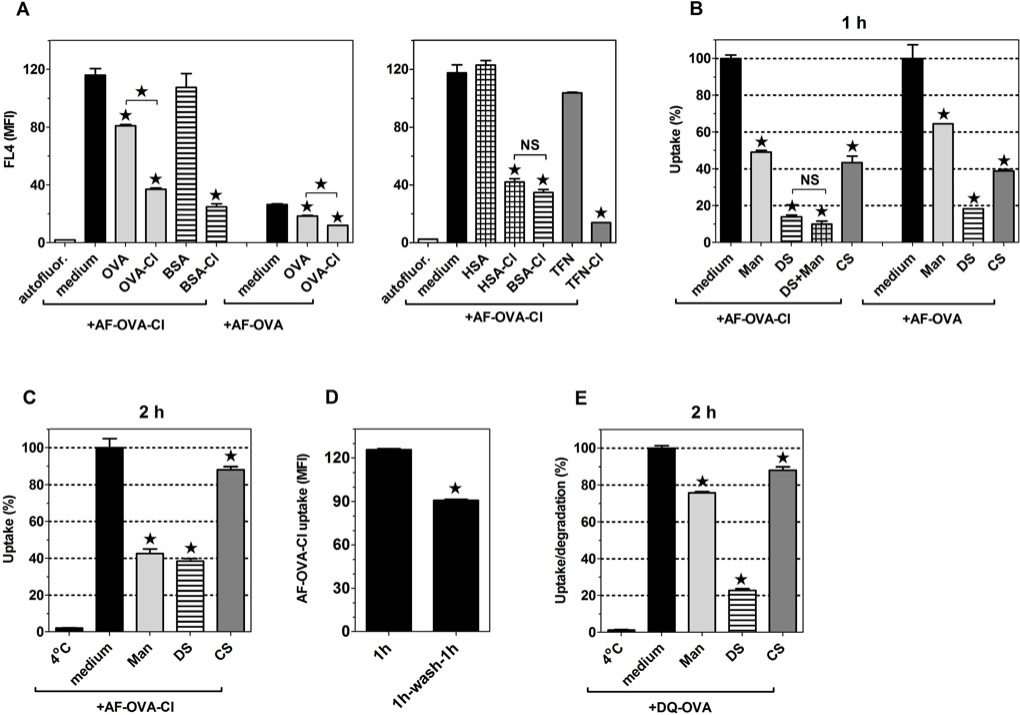
OVA and OVA-Cl bind to the same receptors, which are inhibited by mannan (Man), DS and CS and also shared with other HOCl-modified proteins and glycoproteins. (**A**, **B**, **C**) BM-DC were pre-incubated for 20 min with 1 mg/ml of indicated unlabelled proteins (A), 6 mg/ml Man, 0.2 mg/ml DS or CS (B, C) before the same volume of double-concentrated solution of AF-OVA or AF-OVA-Cl was added to give the final concentration of 5 μg/ml and the incubation was continued for 1 h (A, B) or 2 h (C) in a cell culture incubator. Following washing, cell-associated fluorescence was quantified by flow cytometry. (**D**) BM-DC were incubated for 1 h with 5 μg/ml AF-OVA-Cl, washed and either directly assessed for antigen uptake or incubated for another 1 h in medium alone before the cell-associated fluorescence was measured. (**E**) Following pre-incubation with DS or Man, BM-DC were incubated for 1 h on ice with 20 μg/ml DQ-OVA. Unbound DQ-OVA was washed out and the cells were either directly assessed for DQ-OVA binding (“4°C”) or transfer to 37°C for 2 h before the measurement. Results shown are averages ± SEM of triplicates obtained in single experiments, each repeated at least 3 times with similar results. Statistical analysis was performed with ANOVA, followed by the Tukey-Kramer post-test (A-C, E) or with the Student’s t-test (D). *, p < 0.05; NS, non-significant.

Uptake of both AF-OVA and AF-OVA-Cl was inhibited more strongly by unlabelled OVA-Cl than by unlabelled OVA (Fig 5A, *left panel*). This mutual cross-competition indicates that OVA and OVA-Cl bind to shared receptors, exhibiting higher affinity for OVA-Cl as compared to OVA.

Next, we assessed whether also other chlorinated proteins exhibit increased binding to BM-DC by testing their ability to compete with the binding of AF-OVA-Cl. It has been suggested in a previous report of Prokopowicz et al. that oxidised oligosaccharides present in OVA-Cl are responsible for its increased binding to BM-DC [12]. However, we found that chlorinated bovine serum albumin (BSA-Cl), which is not a glycoprotein, competed effectively with the binding of AF-OVA-Cl, whereas native BSA was ineffective in this regard (Fig 5A). As BSA is a lipid transport protein and oxidised lipids in oxLDL are known to contribute to oxLDL binding to endocytic receptors (SR) [23], there was a possibility that such oxidised lipids are also responsible for increased binding of BSA-Cl to BM-DC. However, fatty acid-free HSA-Cl competed with the binding of AF-OVA-Cl to BM-DC with a similar effectiveness as BSA-Cl (Fig 5A, *right panel*). These results indicate that oxidation of amino acid residues themselves is sufficient to confer on proteins increased binding to endocytic receptors on BM-DC. Also chlorinated human apo-transferrin (TFN-Cl) competed strongly with AF-OVA-Cl binding to BM-DC, whereas native TFN produced little inhibition (Fig 5A, *right panel*).

### Effects of competitive ligands reveal that uptake of OVA and OVA-Cl is mediated by two major classes of receptors

Attempting to identify specific receptors responsible for OVA-Cl uptake by BM-DC, we begun from examining effects of general blockers of major endocytic receptors: mannan, a ligand of MR, DS, a non-selective inhibitor of SR, and of CS, a chemically similar to DS control polyanion which does not bind to SR [24]. Both DS [25] and CS [26] were also reported to be ligands of MR. DS strongly inhibited uptake of both AF-OVA and AF-OVA-Cl (Fig 5B). A weaker inhibition was produced by mannan and CS. In dose-response experiments, mannan at 3 mg/ml and DS at 0.1 mg/ml exerted already maximal effects, corresponding to ~50% and ~90% inhibition of OVA-Cl (5 μg/ml) uptake (Fig 5B). Importantly, the combination of mannan and DS did not inhibit more strongly OVA-Cl uptake than DS alone. These results indicate that OVA-Cl uptake by BM-DC is mediated by two types of receptors: the first one inhibited by both mannan and DS and accounting for ~50% of total OVA-Cl uptake, and the second one, inhibited by DS but not mannan, and responsible for ~40% (90–50) of the uptake.

Interestingly, when BM-DC were allowed to endocytose AF-OVA-Cl for a longer time, for 2 h instead of 1 h, the potency of mannan to inhibit AF-OVA-Cl uptake slightly increased and that of DS strongly decreased (Fig 5C). Among possible explanations of these results is that AF-OVA-Cl undergoes more intense degradation when internalised through receptors inhibited by DS, but not by mannan, as compared to when internalised through receptors inhibited by both competitors. Supporting this interpretation, when BM-DC were first pulsed with AF-OVA-Cl for 1 h and then incubated for an additional hour in medium alone, their fluorescence decreased by ~30%, being indicative of intracellular degradation of endocytosed AF-OVA-Cl (Fig 5D). More intense degradation of ligands internalised through receptors blocked by DS only has been confirmed in experiments with DQ-OVA. Degradation of DQ-OVA was strongly inhibited by receptor blockade with DS, but only by ~24% by mannan (Fig 5E).

### Endogenous, neutrophils-derived HOCl is capable of increasing immunogenicity of proteins

**Fig 6:**
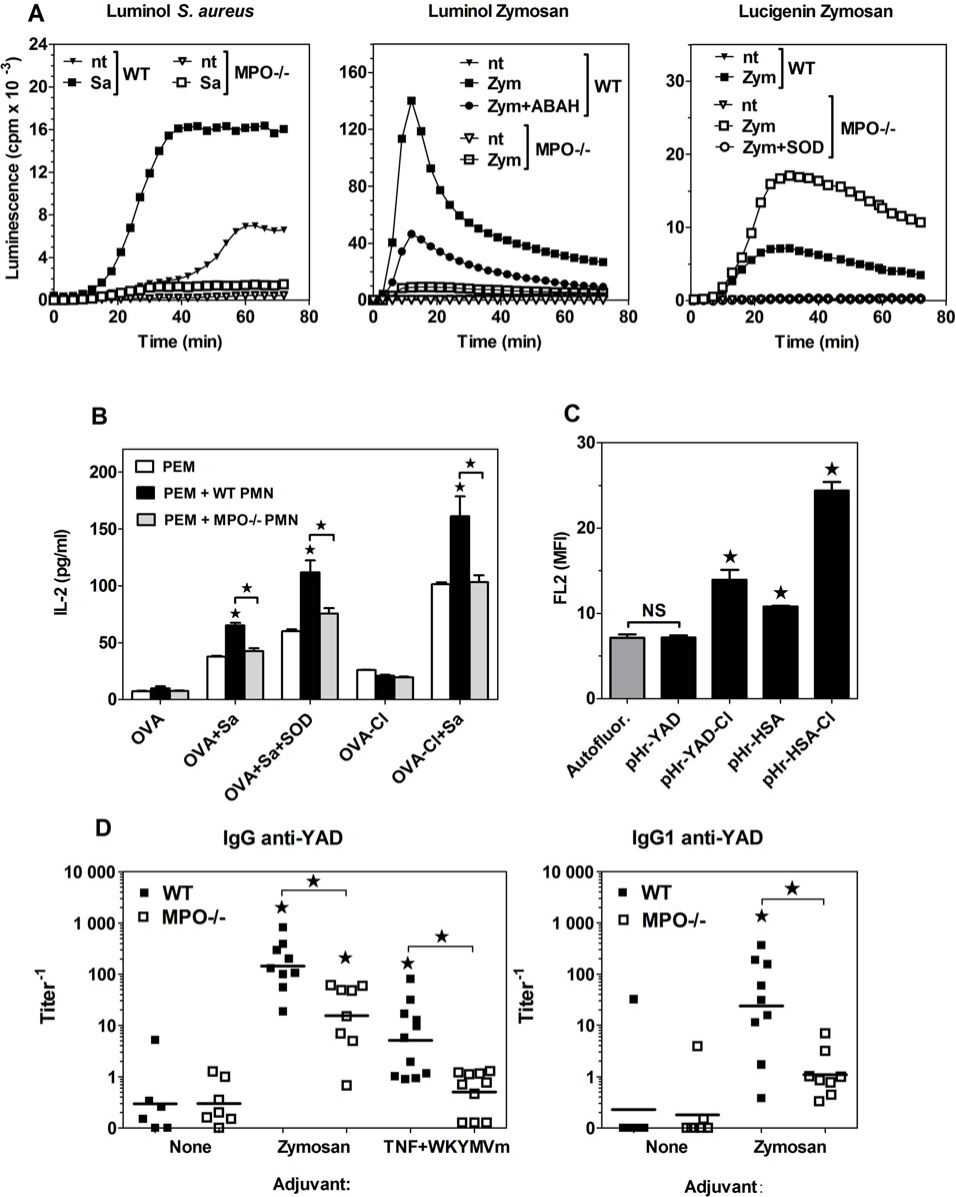
Immunogenicity of proteins is enhanced as the result of oxidation by endogenous, neutrophils-derived HOCl. (**A**) Luminol- or lucigenin-enhanced chemiluminescence stimulated by zymosan (Zym) or heat-killed *S*. *aureus* (Sa) in WT and MPO-/- neutrophils. (**B**) Presentation of OVA, as assessed by IL-2 production, by PEM pre-incubated with OVA, *S*. *aureus* and WT or MPO-/- neutrophils (PMN) to subsequently added CD4^+^ OT-II lymphocytes. (**C**) Uptake of pHrodo-labelled native or HOCl-oxidised YAD or HSA by BM-DC, assessed by flow cytometry. Results of single experiments shown were repeated 2 more times with similar results. (**D**) Titers of YAD-specific IgG in sera of mice primed with 20 μg YAD and hot alkali-treated zymosan or TNF-α plus WKYMVm peptide and boosted 2 weeks later with 20 μg of YAD alone. Sera were collected 8 days after the boost immunization and titers of YAD-specific IgG were determined by ELISA. Points represent titer values in individual mice and horizontal lines geometric means. Statistical analysis was performed with ANOVA, followed by the Tukey-Kramer post-test, to compare all pairs of groups (B, D) or by the Dunnett’s test, to make comparisons with the control group (“Autofluor.”) (C). *, p < 0.05; NS, non-significant.

The results presented above confirm that treatment with HOCl added as a reagent increases immunogenicity of OVA. The purpose of the next series of experiments was to verify the physiological significance of this process, i.e. evaluating the possibility that oxidation by endogenous HOCl, produced by activated neutrophils, leads to increased immunogenicity of proteins. For this purpose, adherent PEM were co-cultured with neutrophils in the presence of native OVA. Production of HOCl in neutrophils was induced by heat-killed *S*. *aureus*. As shown in Fig 6A, both the bacteria and zymosan stimulated ~10-times stronger luminol chemiluminescence in WT than in MPO-/- neutrophils (as estimated by calculating the areas under curves), confirming that chemiluminescence under these conditions depends on MPO activity. Consistently, the zymosan-stimulated luminol chemiluminescence in WT neutrophils was inhibited in ~63% by 0.5 mM ABAH, an inhibitor of MPO. In contrast, both zymosan (Fig 6A, *right panel*) and *S*. *aureus* (S2 Fig) stimulated ~2-fold stronger lucigenin chemiluminescence in MPO-/- than in WT neutrophils. Zymosan-stimulated lucigenin chemiluminescence was quenched completely by 5 kU/ml SOD (Fig 6A, *right panel*), but unaffected by ABAH (S2 Fig), confirming the major role of extracellular O_2_^-^ in generating lucigenin chemiluminescence.

Like BM-DC, PEM presented more effectively OVA-Cl than OVA (Fig 6B). Both OVA- and OVA-Cl-stimulated IL-2 production in the co-culture was enhanced by *S*. *aureus*. The inclusion of WT neutrophils during pre-incubation of PEM with OVA significantly increased IL-2 production by subsequently added Th lymphocytes, but only when *S*. *aureus*, a trigger of respiratory burst, was also present. In contrast, no such effect was produced by MPO-/- neutrophils, despite the above shown, much higher production of O_2_^-^ in these cells. These results indicate that neutrophils are able to convert native OVA to more immunogenic forms and that HOCl is the oxidant responsible for this modification. The role of HOCl is further supported by the enhancing effect on OVA immunogenicity of SOD, an enzyme that depletes O_2_^-^, but increases the level of H_2_O_2_, a substrate in MPO-catalysed HOCl production. Interestingly, WT, but not MPO-/- neutrophils also increased immunogenicity of OVA-Cl (Fig 6B).

In order to assess the role of HOCl-mediated oxidation of proteins in vivo, we immunized WT and MPO-/- mice with 20 μg of native HSA together with hot alkali-treated zymosan as a trigger of neutrophil influx and the respiratory burst in these cells. Two weeks later mice were given, also by *i*.*p*. route, boost immunization with 20 μg HSA alone and sera were collected for Ab determination 8 days later. It turned out that in MPO-/- mice immunization with HSA plus zymosan did not stimulate lower, but rather tended to stimulated higher production of specific Ab than in WT mice (S3 Fig, *top and middle panels*). Similar results were obtained when OVA was used as the antigen (S3 Fig, *bottom panel*). Strong up-regulation of several immune mechanisms in MPO-/- mice have been already reported [27]. We hypothesized that our failure to demonstrate the role of MPO-mediated oxidation of protein antigens in vivo may be caused by the fact that the vertebrate proteins used by us bind to endocytic receptors also in their native forms. We therefore decided to use yeast alcohol dehydrogenase (YAD) as the antigen. As shown in Fig 6C, unlike native HSA and HOCl-oxidised YAD, native YAD was not endocytosed by BM-DC. Immunization with native YAD alone induced no or very low titers of specific IgG in both WT and MPO-/- mice (Fig 6D). However, co-administration of YAD with depleted zymosan induced on average 9.5 (total IgG)– 21.6 (IgG1)-times higher titers of YAD-specific Ab in WT than in MPO-/- mice, confirming that oxidation by neutrophils’ MPO is able to increase immunogenicity of proteins also in vivo. We have suspected that the residual low-level Ab production which has persisted in MPO-/- mice might be caused by the acquisition of proteins by APC as the result of co-uptake together with porous, hollow zymosan particles [28]. We therefore used soluble inducers of acute inflammation—a mixture of TNF-α and the WKYMVm peptide as an adjuvant. Co-administration of this non-phagocytosable adjuvant with YAD enhanced significantly the humoral response only in WT, but not in MPO-/- mice (Fig 6D, *left panel*), demonstrating the critical role of HOCl-mediated oxidation in conferring immunogenicity on exogenous proteins.

### Scavenger receptors A (CD204) and CD36 are responsible for the portion of OVA-Cl uptake inhibited by DS, but not by mannan

**Fig 7:**
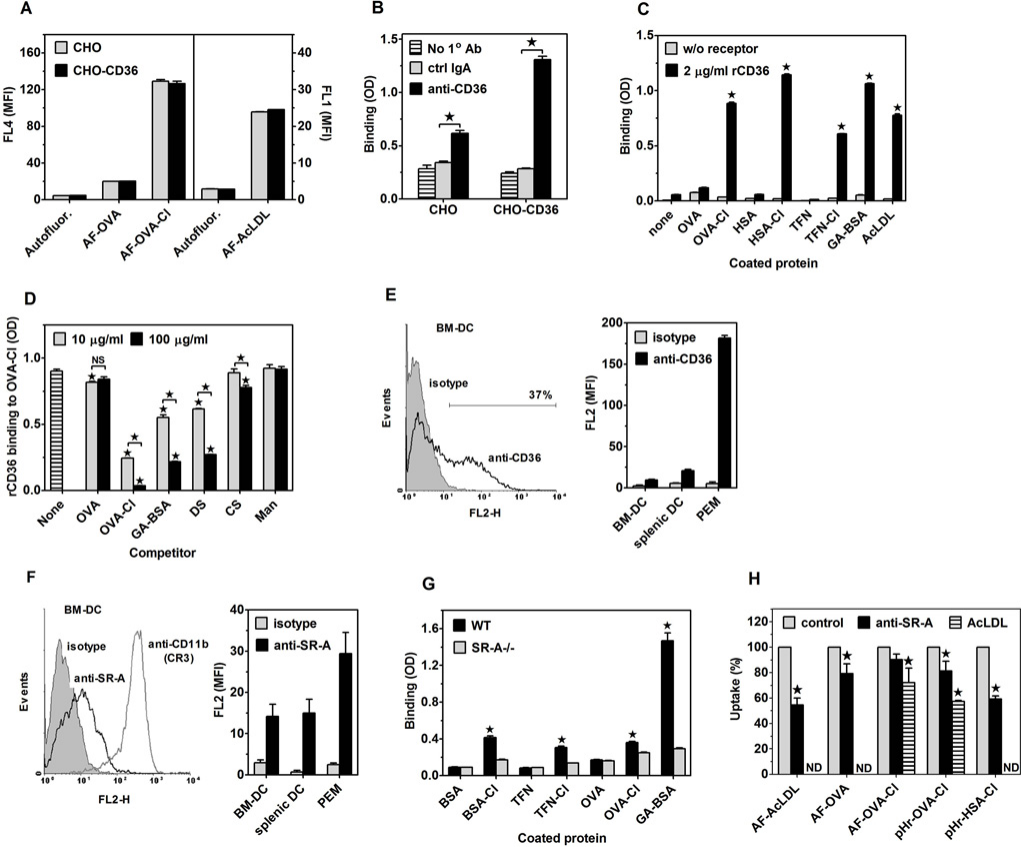
Roles of SR CD36 (A-E) and SR-A (G-I) as receptors for HOCl-modified proteins. (**A**) Uptake of fluorescently-labelled proteins by CHO cells transfected with human CD36 as compared to non-transfected cells. (**B**) Binding of anti-CD36 mAb and control mouse IgA to CD36-transfected and non-transfected CHO cells, determined by cellular ELISA. (**C**) Binding of rCD36 to plate-adsorbed proteins. (**D**) Effects of 10 or 100 μg/ml of indicated, soluble ligands on rCD36 binding to plate-adsorbed OVA-Cl. (**E**) Binding of polyclonal anti-mouse CD36 Ab and control goat IgG to BM-DC, splenic DC and PEM. A representative histogram of Ab binding to BM-DC is displayed on the left graph. (**F**) Binding of anti-mouse SR-A mAb and control rat IgG2b to BM-DC, splenic DC and PEM. A representative histogram of Ab binding to BM-DC is shown on the left graph. (**G**) Binding of SR-A present in lysates of PEM to plate-adsorbed proteins. (**H**) Effects of anti-SR-A 2F8 mAb and AcLDL on the uptake of fluorescently-labelled proteins by BM-DC. Shown are results of single experiments, each representative of at least 3 similar experiments performed (A-D, G, H) or averages +SEM from 4–6 independent experiments (E, F). The data were analysed with the unpaired (A-C, G) or one-sample (H) Student’s t-test or with ANOVA, followed by the Tukey-Kramer post-test (D). *, p < 0.05; ND, not done.

Stronger inhibition of AF-OVA-Cl uptake by DS than by CS suggests an involvement of SR in this process. Amongst them, the class B SR CD36 has been demonstrated to serve as a receptor for HOCl-oxidised LDL [29]. Aiming to examine the role of CD36 as a receptor for OVA-Cl, we compared uptake of AF-OVA-Cl by CHO cells transfected or not with CD36. Surprisingly, non-transfected CHO cells exhibited both the pattern and magnitude of fluorescently-labelled ligands uptake very similar to BM-DC and transfection with CD36 did not have any effect on this uptake (Fig 7A). These results indicate that either CD36 is not a receptor for OVA-Cl or that endogenous receptors of CHO cells are already sufficient to mediate the maximal uptake, which therefore cannot be further increased by overexpression of CD36. Supporting the latter possibility, we detected specific binding of anti-CD36 mAb to non-transfected CHO cells (Fig 7B). Thus, results of experiments with transfected cells are inconclusive regarding the role of CD36 as OVA-Cl receptor. We therefore resorted to a different approach, namely studying binding of isolated rCD36 to proteins adsorbed to ELISA plates. As shown in Fig 7C, rCD36 bound strongly to immobilized GA-BSA and AcLDL, used as positive controls. The binding of rCD36 to OVA-Cl, TFN-Cl and HSA-Cl was as high as to GA-BSA. In contrast, rCD36 did not exhibit specific binding to native proteins. Binding of rCD36 to adsorbed OVA-Cl was dose-dependently inhibited by soluble DS and GA-BSA, but not by CS and mannan (Fig 7D), which confirms that rCD36 retains the ligand binding specificity of natural CD36. Surprisingly, soluble OVA-Cl inhibited rCD36 binding even more potently than DS or GA-BSA, whereas OVA had no effect. These results indicates that upon oxidation by HOCl proteins gain the ability to bind to CD36 with high affinity.

We started verification of CD36 involvement in OVA-Cl uptake by BM-DC from examining CD36 expression on these cells. As shown in Fig 7E, CD36 was expressed in ~36% of BM-DC at low level. At slightly higher level CD36 was also expressed on splenic DC. In contrast, PEM exhibited extremely high expression of CD36; specific binding of goat anti-mouse CD36 Ab to PEM was as much as ~25-fold higher than its binding to BM-DC (Fig 7E). We then assessed the role of CD36 in OVA-Cl uptake with the use of function-blocking anti-CD36 mAb. This Ab had no effect on AF-OVA or AF-OVA-Cl uptake by BM-DC (S4A Fig).

SR-A has been reported to serve as a receptor for numerous covalently-modified proteins [30]. BM-DC were confirmed to exhibit expression of SR-A, which was relatively low as compared to CD11b expression (Fig 7F). The expression of SR-A on splenic DC was similar to that on BM-DC, whereas PEM express ~2-times more SR-A (Fig 7F). In order to examine the role of SR-A as a receptor for HOCl-modified proteins we took advantage from the method developed by Peiser et al. [16], in which binding of SR-A present in lysates of macrophages to plate-adsorbed proteins is examined. As shown in Fig 7G, SR-A bound strongly to GA-BSA, used as a positive control. In comparison, binding of SR-A to HOCl-modified protein was weak, with the strongest one to BSA-Cl, and no binding of SR-A to native proteins was observed. These results indicate that HOCl-oxidised proteins are ligands of SR-A. The role of SR-A in the uptake of HOCl-modified proteins by BM-DC was then assessed with the use of blocking 2F8 mAb. Although in BM-DC this mAb at 20 μg/ml inhibited by ~35% uptake of AF-AcLDL, the prototype ligand of SR-A, it had no consistent effect on AF-OVA or AF-OVA-Cl uptake (S4B Fig). At 2-fold higher, 40 μg/ml concentration anti-SR-A mAb also had no significant effect on AF-OVA-Cl uptake, but inhibited by ~21% uptake of AF-OVA and by ~20% internalisation of pHr-OVA-Cl (Fig 7H). Anti-SR-A mAb produced even stronger, ~41% inhibition of internalisation into acidic compartments of pHr-HSA-Cl, similar to its effect on the uptake of AF-AcLDL (~45%) (Fig 7H). It seems, however, that effects of even this high concentration of anti-SR-A mAb underestimate the contribution of SR-A to the uptake of protein ligands as SR-A deficiency had much stronger effect on these ligands uptake (Fig 3A). Uptake of AF-OVA-Cl and internalisation of pHr-OVA-Cl were inhibited more strongly than by 2F8 mAb by unlabelled AcLDL, by ~28 and 43%, respectively (Fig 7H) and the use of AcLDL seems to enable accurate estimation of SR-A contribution to the uptake of proteins by BM-DC as AcLDL decreased their uptake to the level seen in SR-A-/- BM-DC and had no effect on the uptake by SR-A-/- cells (S4C Fig, *left panel*).

For examining roles of CD36 and SR-A in the presentation of HOCl-modified proteins to specific Th lymphocytes by live APC the available blocking agents could not be used because, as we showed previously [18,31], they exhibit agonistic properties upon binding to receptors and trigger intracellular signalling which affects APC functions of macrophages and DC. We therefore took advantage of the availability of receptor-deficient mice. In comparison to WT controls, in SRA-/- BM-DC uptake of AF-OVA-Cl was decreased by ~27% (Fig 3A, *left panel*). SR-A-deficiency had an even stronger effect on the internalisation of HOCl-modified proteins into acidic endosomes: relative to WT cells, in SR-A-/- cells endocytosis of pHr-OVA-Cl was decreased by ~48% and that of pHr-HSA-Cl by as much as ~65% (Fig 3A, *right panel*). As might be expected, in SR-A deficient BM-DC uptake of AcLDL was very strongly decreased (~80%). BM-DC exhibited minimal endocytosis of pHrodo-labelled native HSA, ~12-times lower than that of the same concentration of pHr-HSA-Cl, which was not significantly affected by SR-A deficiency (Fig 3A, *right panel*). The lack of SR-A in BM-DC had a delayed effect on the presentation of OVA-Cl by these cells, as assessed by IL-2 production in the co-culture with OT-II CD4^+^ lymphocytes: ~28% decrease of IL-2 production relative to the co-culture with WT BM-DC was observed in the 2^nd^, but not in the 1^st^ day of co-culture (Fig 3C). The relatively mild effect of SR-A deficiency on the presentation of OVA-Cl does not seem to be caused by compensatory up-regulation of alternative endocytic receptors, as SR-A-/- BM-DC expressed unaltered levels of MR and SREC-I, whereas expression of CD36 was actually decreased by ~31% on SR-A-/- BM-DC relative to WT BM-DC (Fig 3B).

In CD36-deficient BM-DC uptake of AcLDL was decreased by ~18%, but CD36 deficiency had no significant effect on the uptake of any other ligand tested (Fig 3A). Also, CD36-deficient BM-DC presented OVA and OVA-Cl as effectively as WT cells (Fig 3C). Together with the above shown ineffectiveness of blocking mAb (S4A Fig), these results indicate that CD36 does not have an evident contribution to either uptake or presentation of HOCl-modified proteins by BM-DC, which may be caused by the fact that only a minority of BM-DC express this receptor at low level. Therefore, in order to verify the ability of CD36 to mediate uptake and presentation of HOCl-oxidised proteins we examined PEM, another type of APC which, as shown in Fig 7E, express CD36 at very high level. Relative to WT PEM, in CD36-/- PEM uptake of AF-OVA-Cl was decreased by ~17% and internalisation of pHr-OVA-Cl by ~27% (Fig 3D). In contrast, CD36-deficiency had no significant effect on the uptake of AF-OVA or pHr-HSA-Cl (Fig 3D).

Relative to WT PEM, CD36-/- PEM expressed as much as ~2-times more SR-A on their surfaces (Fig 3E). However, a relatively mild effect of CD36 deficiency on the uptake of OVA-Cl does seem to result from the compensation by enhanced SR-A-mediated uptake because in SR-A-deficient PEM AcLDL or oxLDL had similar effects on AF-OVA-Cl or pHr-OVA-Cl uptake (S4C Fig, *middle and right panels*) as CD36 deficiency (Fig 3D). SR-A was responsible for ~35% of the total uptake of AF-OVA-Cl and ~40% of pHr-OVA-Cl internalisation, as indicated by both effects of SR-A deficiency (Fig 3D) and of AcLDL on these ligands uptake by CD36-/- PEM (S4C Fig, *middle and right panels*). SR-A-deficient PEM also exhibited very strong impairment of AF-OVA (~42%) and pHr-HSA-Cl (~46%) uptake (Fig 3D).

Despite SR-A contributing more to the uptake of OVA-Cl than CD36, CD36-deficient, but not SR-A-deficient PEM exhibited significantly impaired presentation of OVA-Cl: ~39 and 32% decrease of IL-2 production in the co-culture with CD36-/- PEM was observed in the 1^st^ and the 2^nd^ day, respectively (Fig 3F). Our results also confirm superior APC function of DC over macrophages. OVA-Cl-pulsed BM-DC stimulated ~4.4–7.2-times higher IL-2 production in OT-II lymphocytes than PEM and native OVA stimulated significant IL-2 production only in the co-culture with BM-DC (compare Fig 3C and 3F). Moreover, antigen presentation by PEM was more transient than by BM-DC, as indicated by decreased IL-2 levels in the 2^nd^ day when PEM, but not BM-DC were used as APC. However, stimulation with LPS improved presentation of OVA-Cl by PEM (~10.2-fold in the 1^st^ day), more strongly than by BM-DC (~4.3-fold) (Fig 3G). Moreover, whereas LPS abolished the effect of SR-A deficiency on the presentation of OVA-Cl by BM-DC, in PEM it potentiated the effect of SR-A deficiency, which became significant (~41%).

We also assessed effects of CD36 or SR-A deficiencies on the presentation of HOCl-oxidised proteins in vivo (Fig 3H). In comparison to WT controls, titers of antigen-specific IgM were on average ~2.3-times higher in sera of SR-A-/- mice immunized 8 days earlier with 20 μg OVA-Cl, but not with 20 μg HSA-Cl. In contrast, in CD36-/- mice primary humoral responses to HSA-Cl, but not to OVA-Cl, were significantly impaired, as indicated by ~2.5-times lower titers of HSA-specific IgM.

### SR LOX-1 and SREC-I as well as RAGE are able to bind chlorinated proteins, but do not contribute to OVA-Cl uptake by BM-DC

**Fig 8:**
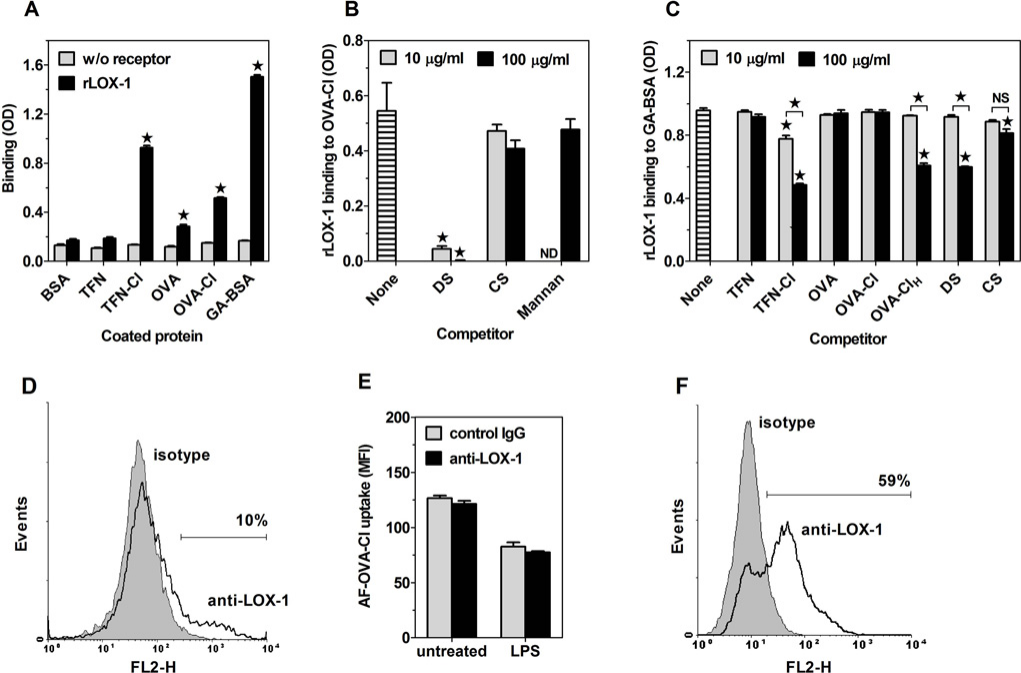
LOX-1 is capable of binding HOCl-modified proteins, but does not contribute to OVA-Cl uptake by BM-DC. (**A**) Binding of rLOX-1 to plate-adsorbed proteins. (**B**, **C**) Effects of 10 or 100 μg/ml of indicated, soluble ligands on rLOX-1 binding to plate-adsorbed OVA-Cl (B) or GA-BSA (C). (**D**) Binding of PE-conjugated anti-mouse LOX-1 mAb and control rat IgG2a to CBA BM-DC, determined by flow cytometry. (**E**) The effect of blocking goat anti-mouse LOX-1 polyclonal Ab, relative to normal goat IgG, on AF-OVA-Cl uptake by untreated and LPS-pre-treated CBA BM-DC. (**F**) LOX-1 expression on LPS-pre-treated CBA BM-DC. Results of single experiments are shown, repeated at least twice with similar results. The data were analysed by the Student’s t-test (A, E) or by ANOVA, followed by the Tukey-Kramer post-test (B, C). *, p < 0.05; NS, non-significant.

The role of LOX-1 as a receptor for HOCl-modified proteins has been suggested by a recent report demonstrating increased uptake of fluorescein-labelled OVA-Cl by CHO cells upon transfection with LOX-1 [12]. As shown in Fig 8A, rLOX-1 bound strongly to GA-BSA, used as a positive control, and only slightly less strongly to TFN-Cl. In comparison, the binding to OVA-Cl was weak, even weaker to OVA and no specific binding of rLOX-1 to BSA or TFN was observed. Binding of rLOX-1 to OVA-Cl was almost completely blocked by DS already at 10 μg/ml (Fig 8B). In contrast, CS and mannan had no effect on rLOX-1 binding to OVA-Cl, indicating that rLOX-1 preserves the ligand binding specificity of natural LOX-1. Results of additional competition experiments have confirmed low affinity of OVA and OVA-Cl for rLOX-1; whereas TFN-Cl dose-dependently inhibited rLOX-1 binding to GA-BSA, no inhibition was produced by OVA or OVA-Cl even at 0.1 mg/ml (Fig 8C). HOCl-modified lipoproteins, shown to bind to human LOX-1, were oxidised with higher concentrations of HOCl [32] than our OVA-Cl preparation. We therefore prepared a more heavily HOCl-oxidised OVA-Cl by incubating OVA with 6 mM instead of 3 mM NaOCl (OVA-Cl_H_), and assessed its binding to rLOX-1. OVA-Cl_H_ turned out to be a higher affinity ligand of LOX-1 than OVA-Cl, inhibiting rLOX-1 binding with a similar potency as DS (Fig 8C). We then assessed involvement of LOX-1 in OVA-Cl uptake by BM-DC. LOX-1 was expressed on ~10–13% BM-DC generated from C57BL/6 or CBA mice (Fig 8D). LOX-1-positive BM-DC were among cells exhibiting the highest level of AF-OVA-Cl uptake (S6A Fig). However, blocking anti-LOX-1 Ab had no effect on AF-OVA-Cl uptake by BM-DC of either strain (Fig 8E). As the apparent lack of LOX-1 involvement in OVA-Cl uptake by untreated BM-DC might be caused by the lack of LOX-1 expression on the majority of cells, we up-regulated LOX-1 expression on BM-DC by pre-incubation with LPS. The LPS pre-treatment increased the average expression level of LOX-1 by as much as 3.4–4.3-fold, and the percentage of LOX-1-expressing BM-DC to ~39%, in case of C57BL/6 cells (S6B Fig), and to ~60%, in case of CBA cells (Fig 8F). However, also in LPS-pre-treated CBA BM-DC blocking anti-LOX-1 Ab had no effect on AF-OVA-Cl uptake (Fig 8E). Collectively, these results indicate that LOX-1 does not function as a receptor for OVA-Cl.

**Fig 9:**
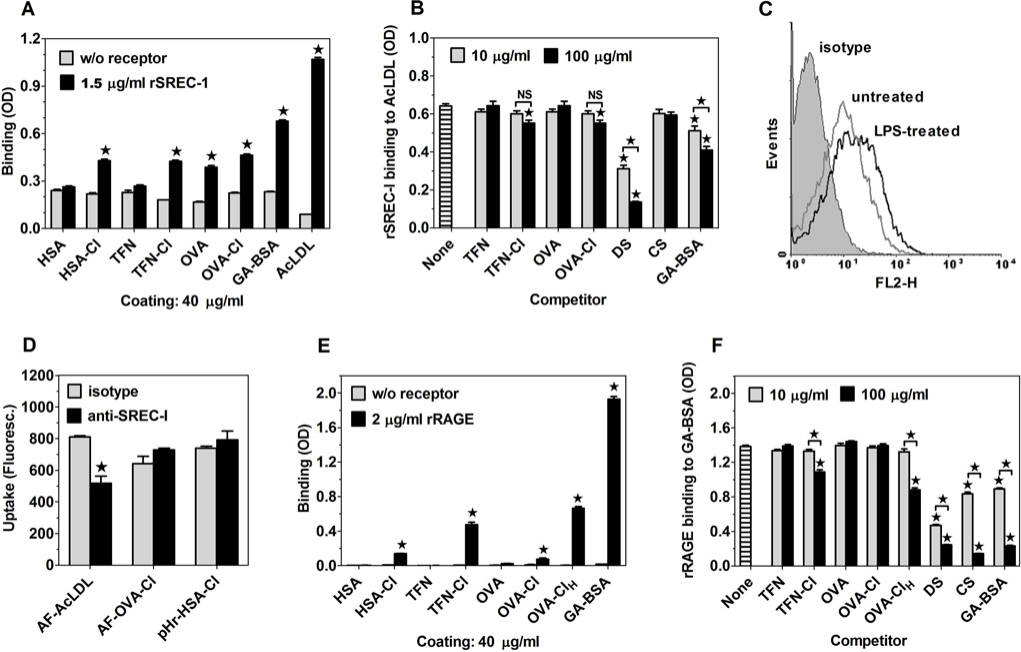
Both SREC-I and RAGE bind HOCl-oxidised proteins with low affinities. (**A**) Binding of rSREC-I to proteins coated onto ELISA plates. (**B**) Effects of 10 or 100 μg/ml of indicated, soluble ligands on rSREC-I binding to plate-adsorbed AcLDL. (**C**) Binding of goat anti-mouse SREC-I Ab to untreated and LPS-pre-treated BM-DC. (**D**) Effects of polyclonal goat anti-mouse SREC-I Ab, relative to normal goat IgG, on the uptake of AF-AcLDL, AF-OVA-Cl and pHr-HSA-Cl by LPS-pre-treated, SR-A-deficient BM-DC. (**E**) Binding of rRAGE to plate-adsorbed proteins. (**F**) Effects of 10 or 100 μg/ml of indicated, soluble ligands on rRAGE binding to plate-adsorbed GA-BSA. The data were analysed by the Student’s t-test (A, D, E) or by ANOVA, followed by the Tukey-Kramer post-test (B, F). *, p < 0.05; NS, non-significant.

Other receptors implicated in the binding of covalently-modified proteins include: SR SREC-I and FEEL-1/stabilin-1 as well as RAGE. From among these receptors, only RAGE was tested for the ability to bind HOCl-oxidised proteins and suggested to be a high-affinity receptor for HOCl-oxidised LDL and BSA, although, in our opinion, the claim concerning affinity of the observed binding was not substantiated [33,34]. When ligands were coated at 20 μg/ml, rSREC-I at 1 μg/ml bound strongly to adsorbed AcLDL and GA-BSA, used as positive controls, but no specific binding to HOCl-modified proteins was observed, except of very weak binding to TFN-Cl (S7A Fig). However, under conditions of increased concentrations of both coated proteins (40 μg/ml) and rSREC-I (1.5 μg/ml), a significant specific binding of rSREC-I to HOCl-modified proteins as well as OVA, but not to TFN or HSA, could be observed (Fig 9A). Low affinity of HOCl-modified proteins for rSREC-I has been confirmed in competition experiments. TFN-Cl and OVA-Cl produced weak, ~14% inhibition of rSREC-I binding to AcLDL only at the higher concentration of 0.1 mg/ml (Fig 9B). In contrast, rSREC-I binding was inhibited strongly and dose-dependently by DS and, less potently, by GA-BSA, but not by CS.

BM-DC exhibited uniform, low expression of SREC-I (Figs 3B and 9C). Two roughly equally numerous populations of PEM differing in size, as indicated by forward scatter, also differed in SREC-I expression. SREC-I was expressed at moderate level on the majority of smaller cells exhibiting high binding of control goat IgG, whereas it was expressed on only ~30% of larger cells to which control goat IgG bound very weakly (S7B Fig). It has been reported that LPS increases SREC-I expression on PEM and, consequently, contribution of SREC-I to AcLDL uptake by these cells [35]. Pre-treatment with LPS increased the cell surface expression of SREC-I on BM-DC ~1.6-fold (Fig 9C). Polyclonal goat Ab, generated against the extracellular portion of mouse SREC-I, inhibited AcLDL uptake by LPS-pre-treated BM-DC by 8–11%, but had no effect in untreated cells (S7C Fig). It has been also reported that SR-A masks the involvement of SREC-I in AcLDL uptake [35]. Consistently, in LPS pre-treated, SR-A-deficient BM-DC anti-SREC-I Ab exhibited increased potency, inhibiting AcLDL uptake by ~36% (Fig 9D). In contrast, the Ab had no effect on AF-OVA-Cl or pHr-HSA-Cl uptake by LPS-pre-treated WT or SR-A-/- BM-DC, indicating that SREC-I does not play a role in the uptake of HOCl-modified proteins by BM-DC. These results corroborate the conclusion drawn from experiments with rSREC-I that HOCl-modified proteins are not high affinity ligands of SREC-I.

In comparison to binding to GA-BSA, used as a positive control, binding of rRAGE to TFN-Cl and OVA-Cl_H_ was weak, and even weaker to OVA-Cl and HSA-Cl (Fig 9E). The same rank of relative ligand affinities for rRAGE was indicated by results of competition experiments. Only the higher, 0.1 mg/ml concentrations of TFN-Cl and OVA-Cl_H_ inhibited rRAGE binding to GA-BSA, by ~21% and ~36%, respectively, whereas OVA-Cl was not inhibitory even at this high concentration (Fig 9F). In contrast, the rRAGE binding was inhibited strongly by DS, GA-BSA and, unlike in the case of SR, also by CS (Fig 9F). Moreover, RAGE was expressed on the surface of neither BM-DC nor PEM, as indicated by the absence of specific binding of three different anti-RAGE Ab: rat anti-mouse RAGE mAb (clone 175440, R&D Systems) and two different goat anti-mouse RAGE polyclonal Ab (from R&D Systems and Santa Cruz Biotechnology) (S7D Fig). The absence of RAGE expression on BM-DC has been reported previously [30]. The lack of specific binding of two different anti-stabilin-1 Ab: goat anti-mouse stabilin-1 and rabbit anti-mouse stabilin-1 polyclonal Ab indicates that also stabilin-1 is not expressed on the surface of BM-DC (S7E Fig).

### The mannose receptor (CD206) is responsible for the component of chlorinated proteins uptake which is inhibited by both mannan and DS

**Fig 10:**
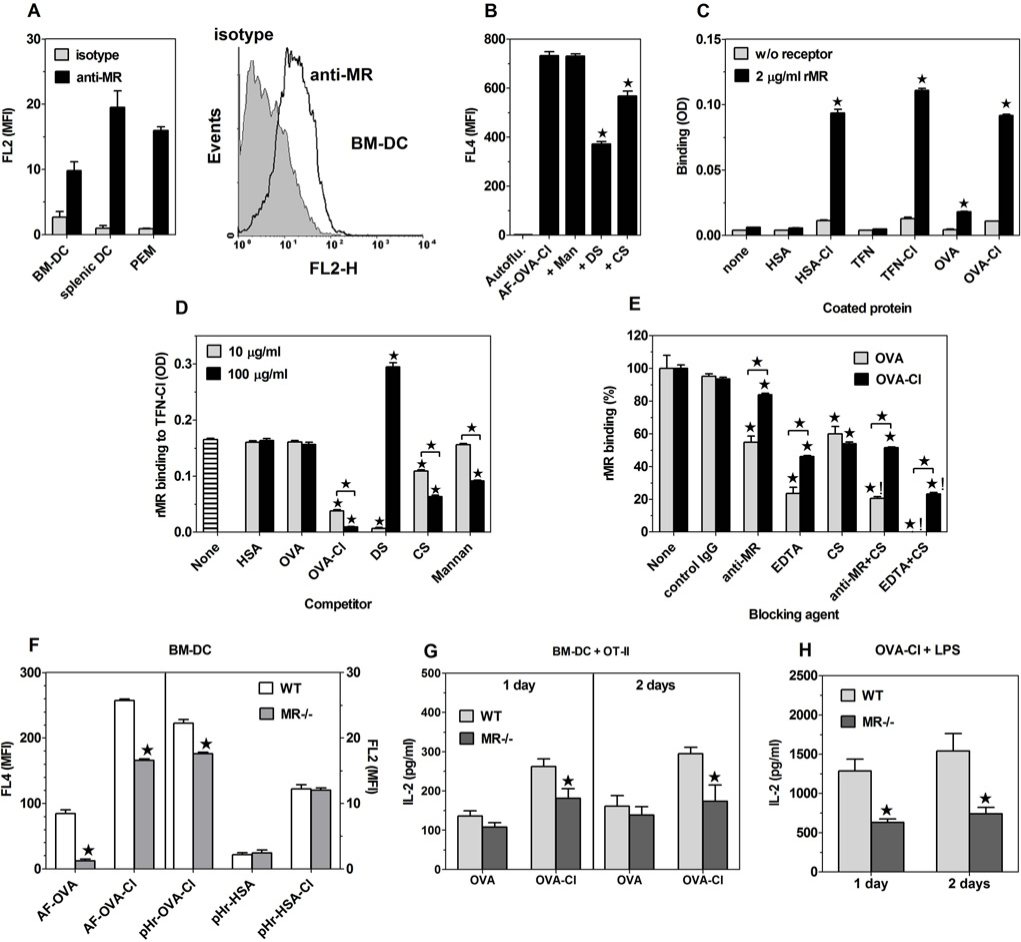
The role of MR as a receptor for HOCl-modified proteins. (**A**) Geometric mean fluorescence intensity of anti-MR mAb and isotype-matched control rat IgG2a binding to BM-DC, splenic DC and PEM. The graph on the right shows a representative histogram for BM-DC. (**B**) Effects of mannan (Man), DS and CS on AF-OVA-Cl uptake by J774 cells. (**C**) Binding of rMR to plate-adsorbed proteins. (**D**) Effects of 10 or 100 μg/ml of indicated, soluble ligands on rMR binding to plate-adsorbed TFN-Cl. (**E**) Effects of agents selectively blocking C-type lectin domains (EDTA, anti-MR mAb) or the cysteine-rich domain (CS) on rMR binding to plate-adsorbed OVA or OVA-Cl. (**F**) Uptake of indicated, fluorescently-labelled ligands by WT and MR-/- BM-DC. (**G**, **H**) IL-2 production in the co-culture of CD4^+^ OT-II lymphocytes with WT or MR-/- BM-DC pulsed for 3.5 h with 20 μg/ml OVA, 7 μg/ml OVA-Cl (G) or OVA-Cl + 200 ng/ml LPS (H). The results shown are averages +SEM from 4 independent experiments (A, F-H) or mean values +SEM obtained in single experiments, repeated 2–3 times with similar results (B-E). The data were analysed by the Student’s t-test (C, F-H) or by ANOVA, followed by the Tukey-Kramer post-test (B, D, E). *, p < 0.05;!, a statistically significant additive effect of two ligands; NS, non-significant.

The above results indicate that SR-A and CD36 are the receptors responsible for the portion of chlorinated proteins uptake which is inhibited by DS, but not by mannan. The best candidate for the receptor mediating the fraction of HOCl-modified proteins uptake which is inhibited by both DS and mannan was the mannose receptor (MR/CD206). First, MR was expressed on BM-DC (Fig 10A). A small parallel shift of the histogram representing cells labelled with anti-MR mAb relative to that depicting cells incubated with an isotype control, indicates that the majority of BM-DC express MR at low level. Splenic DC expressed ~2-times more MR than BM-DC, whereas the expression level of MR on PEM was intermediate (Fig 10A). Second, OVA is a well-established ligand of MR [36], whereas the occurrence of mutual cross-competition indicates that OVA-Cl binds to the same receptors on BM-DC as OVA (Fig 5A). Third, in J774 macrophage-like cells, which express several endocytic receptors at high level, including SR-A [37] and CD36 [17], but do not express MR [38], mannan had no effect on OVA-Cl uptake (Fig 10B). Finally, although mannan is also a ligand of other receptors, MR is the only known receptor that in addition to mannan also binds sulphated saccharides [26].

The ability of MR to bind HOCl-modified proteins has been confirmed in experiments with rMR. rMR bound weakly to adsorbed OVA, used as a positive control (Fig 10C). The binding of rMR to OVA-Cl was as much as ~6-times higher than that to OVA. rMR exhibited similarly high binding to TFN-Cl and HSA-Cl, but no specific binding to HSA and TFN could be detected (Fig 10C). To further characterize binding specificity of rMR, we performed competition experiments. Among non-proteinaceous ligands, DS turned out to be the most potent competitor, essentially blocking binding of rMR to TFN-Cl already at 10 μg/ml, followed by CS and mannan (Fig 10D); mannan and CS inhibited binding of rMR to adsorbed OVA-Cl with IC_50_ values of ~184 and 9.9 μg/ml, respectively (S8A Fig). Whereas OVA-Cl inhibited binding of rMR to TFN-Cl almost as potently as DS, OVA had no effect even at 0.1 mg/ml, confirming that OVA is a low-affinity ligand of MR. However, the affinity of OVA, as well as of other proteins, to MR is dramatically increased by HOCl-mediated oxidation. Paradoxically, DS at 100 μg/ml increased binding of rMR to TFN-Cl (Fig 10D and S8B Fig). At these high concentrations (>10 μg/ml) DS seems to enhance non-specific binding of rMR as it also produced strongly increased binding of rMR to non-coated (blocked only) wells. In contrast, at 10 μg/ml DS selectively inhibited rMR binding to wells coated with TFN-Cl or OVA-Cl, but had no effect on non-specific attachment to uncoated wells (S8C Fig).

Three different types of domains have been implicated in ligands binding to MR: eight C-type lectin domains (CTLD) bind in a calcium-dependent manner oligosaccharides terminating with mannose, fucose or N-acetylglucosamine (with the most prominent role played by CTLD4), the single fibronectin type II repeat is responsible for binding to collagens and the N-terminal cysteine-rich domain (CysR) mediates the receptor binding to sulphated saccharides [26,36,39–42]. In order to gain insight into the role of these domains in MR binding to OVA-Cl, we used domain-selective agents in competition experiments: a divalent cations chelator EDTA or mAb generated against CTLD4-7 to inhibit CTLD and CS to selectively block CysR of MR [26]. Both EDTA and anti-CTLD mAb produced stronger inhibition of rMR binding to OVA (by ~76 and 40%, respectively) than to OVA-Cl (54 and 9%) (Fig 10E), indicating that CTLD play a relatively more important role in MR binding to OVA than to OVA-Cl. Surprisingly, CS inhibited rMR binding to both OVA and OVA-Cl (40 and 46%). However, CS and anti-CTLD mAb had an additive effect on rMR binding to OVA, but not to OVA-Cl, and the combination of CS and EDTA produced complete inhibition of rMR binding to OVA, but only partial, 77% inhibition of the receptor binding to OVA-Cl (Fig 10E). Thus, both CTLD and CysR seem to contribute to OVA and OVA-Cl binding to MR. These two domains account entirely for rMR binding to OVA, whereas in the case of OVA-Cl other parts of MR molecules may be also involved. The complete inhibition of rMR binding to TFN-Cl by DS (Fig 10D) indicates that DS blocks both CTLD and CysR.

The role of MR in the uptake and presentation of HOCl-oxidised proteins has also been assessed in experiments employing BM-DC generated from MR-deficient mice. In comparison to WT controls, in MR-/- BM-DC uptake of AF-OVA-Cl was decreased by ~36% and that of AF-OVA by as much as ~85% (Fig 10F). Effects of MR deficiency indicate that, inversely to SR, MR had a relatively larger contribution to the total uptake of OVA-Cl than to the internalisation of this ligand into acidic endosomes, as in MR-/- BM-DC internalisation of pHr-OVA-Cl was reduced by ~21% only. Internalisation of pHr-HSA and pHr-HSA-Cl was not affected significantly by MR deficiency.

In MR-/- BM-DC presentation of OVA-Cl was decreased to a similar extent as the uptake of AF-OVA-Cl, by ~31 and 41%, respectively, in the 1^st^ and the 2^nd^ day of co-culture (Fig 10G). In contrast, despite the fact that in MR-/- BM-DC uptake of OVA was drastically decreased (Fig 10F), MR-deficient BM-DC did not exhibit significant impairment of OVA presentation (Fig 10G). In MR-/- BM-DC, also the presentation of OVA-Cl in the presence of LPS was decreased, by ~51% (Fig 10H).

### LPS and SR-A ligands modulate intracellular routing and proteolytic processing of endocytosed proteins

**Fig 11:**
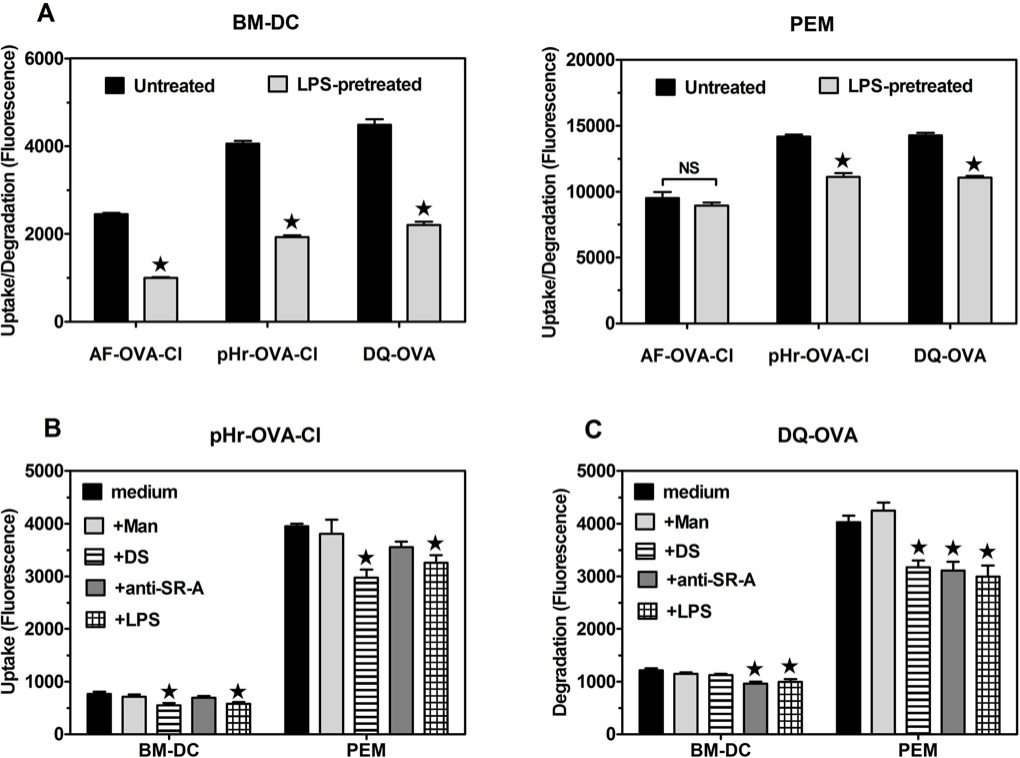
LPS and SR-A ligands regulate uptake, acidification and proteolysis of endocytosed antigens. (**A**) Effects of 1-day pre-treatment with 100 ng/ml LPS on the total uptake of AF-OVA-Cl, internalisation of pHr-OVA-Cl and degradation of DQ-OVA by BM-DC and PEM. (**B**, **C**) Effects on indicated ligands on the acidification of pHr-OVA-Cl-containing endosomes (B) and proteolytic digestion of DQ-OVA (C) in BM-DC and PEM. The results shown are averages +SEM from 3 independent experiments, each performed in 4 replicates. The data were analysed by the Student’s t-test (A) or by ANOVA, with the Dunnett’s post-test applied to compare the control (“medium”) with other groups (B, C). *, p < 0.05.

In addition to the up-regulation of co-stimulatory molecules expression and induction of cytokine production in APC, the adjuvant effect of TLR agonists has also been suggested to involve stimulation of antigen processing [43]. The overnight pre-treatment with LPS strongly inhibited, by ~59%, uptake of AF-OVA-Cl by BM-DC (Fig 11A). In LPS-pre-treated BM-DC acidification of internalised pHr-OVA-Cl and degradation of DQ-OVA were less strongly decreased, by ~51–52%, indicating that in LPS-pre-treated BM-DC strongly decreased uptake of antigens is accompanied by their slightly (by ~20%) enhanced lysosomal degradation. In contrast, in PEM pre-treatment with LPS did not affect the uptake level of AF-OVA-Cl, but decreased by ~23% both pHr-OVA-Cl delivery into acidic endosomes and proteolytic degradation of DQ-OVA (Fig 11A).

We also assessed the effect of an “acute” treatment with LPS on the degradation of internalised antigens. APC were incubated on ice with fluorescently-labelled OVA preparations. Subsequently, unbound antigens were washed out and the cells were transferred to 37°C for 2-h incubation with or without LPS. In BM-DC the LPS treatment inhibited acidification of pHr-OVA-Cl by ~25% (Fig 11B) and degradation of DQ-OVA by ~18% (Fig 11C). Although in PEM LPS had a smaller percent effect on pHr-OVA-Cl delivery into acidic endosomes (~17%), due to the fact that PEM internalised ~5.1-times more pHr-OVA-Cl than BM-DC, the absolute value of inhibition was ~3.5-fold larger (Fig 11B). In contrast, LPS produced ~1.4-times stronger, ~26% inhibition of DQ-OVA degradation in PEM than in BM-DC (Fig 11C). Taking into account that in PEM degradation of DQ-OVA was ~3.3-times more intense, this means that in PEM ~5-times more DQ-OVA was spared from degradation as the result of LPS treatment than in BM-DC. Effects of LPS on DQ-OVA degradation were mimicked by ligands of SR-A, but not by mannan. Inclusion of DS in the incubation medium inhibited delivery of pHr-OVA-Cl into acidic compartments in both PEM (~25%) and BM-DC (~28%) (Fig 11B), whereas proteolytic degradation of DQ-OVA was inhibited by DS in PEM only (~21%) (Fig 11C). In contrast, cross-linking of SR-A with specific mAb inhibited degradation of DQ-OVA in both PEM (by ~23%) and BM-DC (~31%) (Fig 11C), but had no effect on the acidification of pHr-OVA-Cl-containing endosomes (Fig 11B).

## Discussion

During primary immune response, endocytic receptors from SR and the C-type lectin families play the main role in antigen uptake by APC. Paradoxically, these receptors do not bind the majority of native proteins. For instance, the repertoire of proteins bound to MR is limited to collagens and glycoproteins containing oligosaccharides terminated with mannose, fucose or N-acetylglucosamine or modified with sulphuric acid [36,39–42,44], whereas SR-A binds with high affinity to proteins which, owing to covalent modification, gain a net negative charge [45]. During repeated contact with the same antigen, the presence of specific Ab may enable uptake of proteins through Fc receptors, and in case of Ab activating complement, also through complement receptors on APC [46]. Moreover, DC and macrophages were demonstrated to be capable of taking over through SR-A protein antigens bound to BCR on B lymphocytes [47], but also this mechanism is unlikely to play a significant role during primary response, when the percentage of naive B lymphocytes expressing BCR specific for a given antigen is very low. In case of intracellular proteins, their uptake by APC may be facilitated by association with heat shock proteins (HSP). When released from necrotic cells, such complexes of HSP with peptides may be endocytosed by APC through SR and efficiently presented [48].

None of the above listed mechanisms is likely to operate during the first encounter with exogenous protein antigens. In fact, insufficient uptake by APC may be the major limiting factor responsible for the observed very poor immunogenicity of purified proteins. However, it is possible to boost immunogenicity of proteins by co-administration of adjuvants. A shared feature of different types of adjuvants is their ability to trigger acute inflammation, characterized by early influx of activated neutrophils, eosinophils and monocytes [6–8]. Acute inflammation is also triggered by other factors disturbing tissue homeostasis, such as infection and sterile injury. Similarly, parasitic worms, by moving and feeding inside tissues cause damage, provoking inflammatory responses. Chemoattractants, responsible for the influx of neutrophils, at higher concentrations present in inflammatory foci, also stimulate respiratory burst in these cells, which may be further enhanced by pathogens or their products. We demonstrated herein that the potent oxidant produced under these conditions—HOCl converts different proteins and glycoproteins, which in their native forms do not bind or bind weakly to endocytic receptors, into high-affinity ligands of these receptors. Endocytosed proteins are subsequently processed and presented to Th lymphocytes. Importantly, HOCl-modified proteins alone, devoid of contamination with PRR agonists, were able to elicit adaptive immune responses. These results indicate that when the uptake of protein antigens is improved, resulting in the presentation of sufficiently high density of complexes of epitopes with MHC-II on the surface of APC, other factors, such as stimulation of PRR, are not necessary to induce these responses. This conclusion is supported by previously reported observations that selective targeting of protein antigens to either MR or CD36 on DC is sufficient to induce robust immune responses in vivo, in the absence of any adjuvant [4,49]. Results of our experiments aimed at elucidation of mechanisms of induction of immune responses under these conditions have revealed that the TCR-mediated recognition of complexes of non-self oligopeptides with MHC-II by Th lymphocytes alone seems sufficient to induce production of IL-2 in these cells. In turn, a reverse signalling stimulated in BM-DC by engagement of MHC-II up-regulates expression of CD40 in these cells. Subsequent interaction of CD40 on DC with CD40L on Th cells leads to up-regulation of CD86 expression on DC, whereas in Th cells it provides co-stimulation for IFN-γ production. It has been established that differentiation of IFN-γ-producing Th1 lymphocytes is favoured by high density of MHC-II with peptides on the surface of APC [50], which may explain why BM-DC pulsed with OVA-Cl stimulated much higher IL-2 and IFN-γ production than those pulsed with OVA, despite similar production of IL-12p40. The density of epitopes displayed on APC seems to be also the major factor regulating the Th1/Th2 polarisation of immune responses to HOCl-oxidized proteins in vivo, as in CBA mice humoral immune response to a low dose (10 μg) of OVA-Cl_L_ was Th2-polarized, whereas that stimulated by a high dose (100 μg) of the same antigen exhibited the Th1 polarisation. In contrast, confirming our previous report [18], neither OVA nor OVA-Cl stimulated production of IL-4 in the in vitro co-cultures with OT-II CD4^+^ cells under any conditions. This strong bias of immune responses toward the Th1 type in OT-II transgenic mice has also been recently reported by others [51].

We were surprised to find that the ability to bind HOCl-oxidised proteins was a shared feature of all non-homologous and structurally diverse receptors examined by us. However, uptake and presentation of OVA-Cl by BM-DC were mediated by MR and SR-A, whereas in the case of PEM CD36 was additionally involved. Other receptors capable of binding HOCl-oxidised proteins were not involved in the uptake of OVA-Cl by BM-DC because they were either not expressed (RAGE) or required stronger oxidation of OVA (LOX-1, RAGE). As antigenicity of such heavily oxidised proteins is likely to be largely lost, the latter receptors might be involved in the scavenging, but not in the presentation of oxidised proteins. The presence of such heavily oxidised LDL, containing modifications occurring at HOCl:protein molar ratios higher than 100:1, has been detected in human atherosclerotic lesions [52]. It has been established that amino acid side chains are major targets in HOCl-mediated oxidation of proteins, with the order of reactivity being: Met > Cys >> His > Trp > Lys >> Tyr~Arg. As sulphur-containing groups of Met and Cys react with HOCl with by far the fastest kinetics, they become selectively oxidised at very low molar ratios of HOCl to accessible reactive groups in proteins, after which ε-amino groups in Lys side chains react, resulting in the production of chloramines [9,12,53,54]. Oxidation of Met and Lys residues, which are absent in the epitope recognized by OT-II lymphocytes, but common in other OVA epitopes, may explain why the in vivo immunogenicity of OVA-Cl is largely lost, whereas its presentation in the co-culture with OT-II lymphocytes continues to increase even further upon an additional oxidation by neutrophils. Neutralization of the positive charge of ε-amino groups in Lys upon chlorination confers on proteins a net negative charge, being the shared feature of ligands of SR, RAGE and of CysR in MR. Subsequent decomposition of Lys chloramines may lead to the generation of carbonyl products, but in OVA-Cl only ~14% of Lys seemed to undergo such irreversible modifications, as indicated by changes in degrees of labelling with amino-selective fluorescent dyes (thiosulphate, added before labelling, converts chloramines back to amines) and by direct quantification of free carbonyl groups [12]. However, even such low level of modification turned out to be sufficient to transform proteins into high-affinity ligands of CD36 and MR. Consistently, it has been found that HOCl-mediated oxidation of only ~5% of Lys in LDL leads to enhanced recognition by CD36 [29]. In contrast, LDL binding to SR-A requires modification of >16% of Lys, although modification of as many as >60% of Lys was necessary to attain maximal binding [29,55,56]. Nevertheless, despite the fact that proteins oxidised with 3 mM HOCl bound weekly to SR-A (Fig 7G), their uptake by APC was strongly SR-A-dependent. This apparent discrepancy may be explained by the relatively high expression of SR-A (BM-DC) or by SR-A being much more efficient than CD36 in mediating endocytosis of bound ligands (PEM) [56]. Even stronger oxidation was required to render OVA a ligand of RAGE. Whereas generation of SR ligands requires modification of specifically Lys side chains [55], conferring on a protein a strong, net negative charge by itself seems to be sufficient to convert it into a RAGE ligand [57]. Unlike OVA-Cl, TFN-Cl was a high affinity ligand of LOX-1, but we were unable to assess the ability of LOX-1 to mediate presentation of TFN-Cl because of the presence of ubiquitous receptors for native TFN. Consequently, immunization with native human TFN induced very high titer of TFN-specific Ab in mouse sera, which was similar to that induced by TFN-Cl (S9 Fig). Importantly, no detectable Ab production was induced by mouse TFN-Cl, which does not support the suggested possibility [58] that chlorination is able to brake tolerance to self proteins.

Results of experiments with DQ-OVA and comparisons of effects of receptor deficiencies on the internalisation of pHr-OVA-Cl into acidic endosomes *versus* the total uptake of AF-OVA-Cl indicate that proteins internalised through SR are subjected to stronger acidification and more intense degradation than those endocytosed through MR. It has been already reported that in BM-DC and macrophages MR and SR deliver internalised ligands into distinct intracellular compartments [59]. In these cells OVA internalised through MR was directed into stable, early endosomes, which resisted strong acidification for a prolong period, and in which OVA-derived peptides were loaded onto MHC class I molecules for subsequent cross-presentation to CD8^+^ T lymphocytes. In contrast, in macrophages unidentified receptors inhibited by polyinosinic acid, presumably SR, mediated internalisation of OVA into MHC-II-positive endosomes, which underwent rapid acidification and fusion with lysosomes. Moreover, more intense and complete degradation of endocytosed proteins inside macrophages than in DC may be an important reason why macrophages underperformed as APC, despite endocytosing several-fold higher amounts of proteins than BM-DC [60] (S5 Fig). Consequently, whereas in BM-DC a portion of proteins endocytosed through SR-A seems subjected to partial degradation into oligopeptides suitable for loading onto MHC-II, in PEM proteins internalised through the same receptor may undergo faster and more complete degradation, precluding their presentation. However, the excessive degradation of antigens in macrophageal endosomes may be reduced upon activation with LPS and/or IFN-γ [61,62]. Accordingly, we have observed that pre-treatment with LPS inhibits acidification of pHr-OVA-Cl-containing endosomes as well as proteolysis of DQ-OVA in PEM, but not in BM-DC, and more strongly enhances presentation of OVA-Cl-derived peptides by PEM than by BM-DC. Moreover, in macrophages LPS enhanced the effect of SR-A deficiency on the presentation of OVA-Cl, which may also be explained by inhibitory effects of LPS on the digestion of endocytosed antigens. Thanks to this activity LPS may prevent complete proteolytic destruction of a portion of antigens internalised through SR-A, enabling their presentation. Thus, adopting the function of professional APC by macrophages upon recognition of pathogen-associated molecular patterns through their PRR seems to include sparing from total proteolysis and presentation of a larger portion of endocytosed antigens. Interestingly, the effect of LPS on antigen degradation was reproduced by SR-A cross-linking with mAb, likely mimicking SR-A engagement during phagocytosis mediated by this receptor.

Conversely, LPS abolished the effect of SR-A deficiency on the presentation of OVA-Cl by BM-DC. The most likely explanation of this observation is the enhancement of signal transduction from TLR occurring in SR-A-deficient DC. In SR-A-deficient DC [63,64], but not in SR-A-deficient macrophages [18,65], LPS have been reported to stimulate much higher production of pro-inflammatory cytokines than in WT cells as well as upon stimulation with TLR agonists SR-A-/- DC [66], but not SR-A-/- PEM [18], exhibited strongly increased potency in priming adaptive immune responses. Results of Yu et al. [64] indicate that in WT DC intracellular SR-A inhibits TLR4 signalling as the result of binding to TNF receptor-associated factor 6 (TRAF6) and sequestering it in late endosomes/lysosomes. Importantly, SR-A-/- BM-DC also exhibited enhanced T cell stimulatory activity during presentation of protein antigens alone, in the absence of PRR agonists [66]. The mechanism of this effect is unclear, but it may include potentiation of CD40 signalling, which also depends on TRAF6 [67]. Thus, the effects of SR-A deficiency on antigen presentation in vitro and on humoral immune responses in vivo observed in our study may be resultant of two opposite processes: decreased density of peptide-MHC-II complexes on the surface of APC caused by decreased uptake and the relief from immunosuppression exerted in WT DC by intracellular SR-A. The net effect of these opposing factors may be determined by the degree of SR-A-dependency of a given antigen uptake by DC.

Three factors: the use of vertebrate proteins which bind to endocytic receptors on APC also in their native forms, strong enhancement of immune responses occurring in MPO-/- mice, and co-uptake of proteins together with phagocytosed zymosan by APC seemed to be the main reasons of the initial failure of our attempts to demonstrate the role of MPO-mediated protein oxidation in vivo. Accordingly, the microbial protein ­ YAD was not endocytosed by APC in its native form and soluble inducers of acute inflammation significantly enhanced Ab production in response to immunization with YAD in WT, but not MPO-/- mice, confirming in vivo role of proteins oxidation by neutrophils’ MPO in increasing their immunogenicity.

Although increased immunogenicity of HOCl-modified proteins apparently results mainly from enhanced uptake by APC, this does not seem to be the sole mechanism. Prokopowicz et al demonstrated that chlorination strongly increases susceptibility of OVA to digestion by proteases [12]. The strong resistance of native OVA to digestion demonstrated in that study may explain our observation that despite endocytosing ~7-times less OVA, MR-/- BM-DC presented it to CD4^+^ T lymphocytes as effectively as WT cells. Similar results were reported previously by Burgdorf et al. [59]. Apparently, in a weakly-digestive microenvironment of early endosomes, to which ligands internalised through MR are targeted, native OVA is not processed for presentation by MHC-II. However, this weakly acidic microenvironment of early endosomes seems to provide favourable conditions for partial digestion of HOCl-oxidized OVA into fragments suitable for loading onto MHC-II, as in MR-deficient BM-DC presentation of OVA-Cl was decreased to the same extent (31–41%) as the total uptake of AF-OVA-Cl (36%). In the case of BM-DC, also a portion of OVA-Cl internalised through SR-A appears to be presented, whereas in PEM faster and more intense degradation of OVA-Cl internalised through the same receptor seems to largely preclude its presentation. In contrast, in the latter cell type, internalisation through CD36 seems to provide close to optimal conditions for antigen presentation as CD36 deficiency in PEM had much stronger effect on the presentation of OVA-Cl (32–39%) than on the total uptake of AF-OVA-Cl (17%). Consistent with the specialization of CD36 in the presentation of endocytosed antigens, despite high redundancy of receptors for HOCl-oxidized proteins, in CD36-/- mice immunization with HSA-Cl induced significantly lower production of HSA-specific IgM than in WT controls.

**Fig 12:**
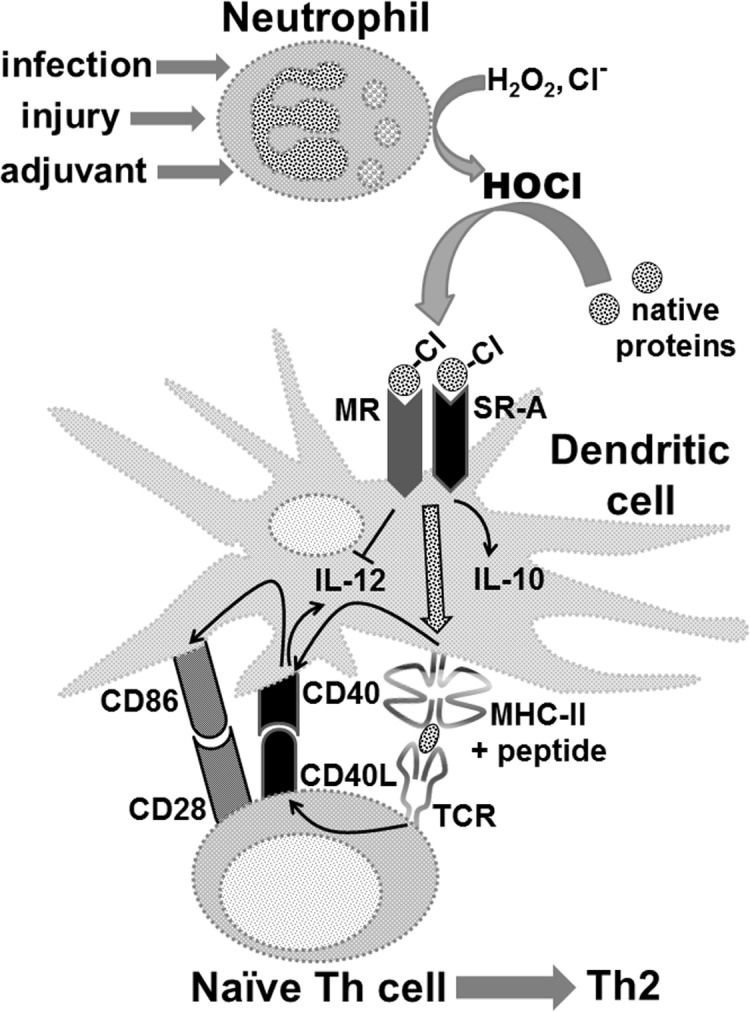
The proposed mechanism of the immunoenhancing effect caused by HOCl-mediated oxidation of protein antigens. Administration of adjuvants, infection or sterile injury trigger acute inflammation, characterized by recruitment and activation of neutrophils. Activated neutrophils produce HOCl which causes non-selective oxidation of both self and, if present, pathogen-derived proteins, subsequently endocytosed by DC through MR and SR-A, processed and presented as complexes with MHC-II on their surface. TCR-mediated cognate interactions of Th lymphocytes with peptide-MHC-II complexes on DC induce IL-2 production in Th lymphocytes (not shown) and up-regulate expression of CD40 on DC and of CD40L on lymphocytes. Upon ligation with CD40L, CD40 induces expression of CD86, a ligand for CD28 on Th lymphocytes. In the absence of PRR ligands, presentation of low density of peptide-MHC-II complexes on DC stimulates differentiation of naive Th lymphocytes towards Th2 cells. The Th2 polarization is reinforced by intracellular signalling triggered upon binding of HOCl-oxidised proteins to SR-A or MR, leading to the suppression of IL-12 and enhancement of IL-10 production by DC.

In summary, our results have revealed a mechanism of recognition of non-self proteins which is accomplished by co-operation between innate and adaptive immunity. We postulate that this mechanism may enable the immune system to detect infections caused by parasitic worms and possibly also by protozoan parasites, which, as being biochemically very similar, do not synthesize kinds of compounds absent in mammalian hosts, and therefore cannot be detected by innate immune cells through their PRR [68,69]. Main biochemical differences between mammalian hosts and these parasites seem to occur in amino acid sequences of proteins, but proteins of all eukaryotic organisms are built from the same set of amino acids, and as such are not good candidates for PRR ligands. However, in the detection of differences in amino acid sequences of proteins lymphocytes, the major cell type of adoptive immunity, are specialized, thanks to being furnished with recombined immunoglobulin receptors. Consequently, one may speculate that infections caused by protozoan and metazoan parasites might be an important part of selection pressure responsible for the evolving of adaptive immunity in jawed vertebrates. The first step in our proposed mechanism of recognition of non-self proteins is induction of acute inflammation, being a non-specific response triggered by essentially any disturbance of tissue homeostasis (Fig 12). One of the major goals of acute inflammation seems to be the recruitment of professional phagocytes. According to the model proposed, HOCl produced by these phagocytes would cause non-selective oxidation of both self and, if present, pathogen-derived proteins, subsequently endocytosed by APC through several endocytic receptors and presented on their surfaces as complexes with MHC molecules. In case of sterile injury, presentation of self proteins only by APC would not induce adaptive immune responses, whereas endocytic receptors, by scavenging apoptotic cells and tissue debris and by regulating production of growth factors and cytokines, would promote resolution of inflammation, immunosuppression and tissue repair [70,71]. In contrast, strongly increased affinity of non-self proteins to endocytic receptors, resulting from their HOCl-mediated oxidation, would allow the immune system to detect their even very small amounts. In the absence of PRR ligands, the major factor determining the type (polarisation) of adoptive immune response may be the dose of antigens. Presentation of high density of complexes of MHC-II with non-self peptides by APC, which either phagocytosed or became infected with intracellular protozoa, would result in the induction of Th1-polarised immune responses, characteristic of infections caused by this type of pathogens [72,73]. In turn, the presentation of only low density of MHC-II-peptide complexes would favour the Th2/Treg polarisation of adoptive immunity during infections with parasitic worms. The Th2/Treg polarisation under these conditions might be reinforced by intracellular signalling triggered upon ligation of endocytic receptors, leading to suppression of IL-12 and IL-6 and stimulation of IL-10 production and therefore creating a cytokine milieu favouring differentiation of Th2/Treg over Th1/Th17 lymphocytes. This possibility is consistent with the observed in this study inability of LPS to reverse the Th2 polarisation of immune responses to HOCl-oxidised proteins and supported by results of our previous studies on signalling abilities of endocytic receptors. We have demonstrated that upon ligation with specific Ab, DS or AcLDL SR-A mediates inhibition of LPS-stimulated IL-6 and IL-12, but enhancement of IL-10 production [18,31], whereas ligation of CD36 with Ab was sufficient alone to induce very high production of IL-10, capable of inhibiting IL-6 and IL-12 production in PEM [18]. Others reported that also MR mediates inhibition of IL-12 and enhancement of IL-10 release [74,75]. In this context, our observation that, unlike in the case of OVA-Cl, increasing the dose of HSA-Cl failed to produce the Th1-polarization of humoral response might be explained by binding of HSA-Cl to SR-A with a higher affinity than of OVA-Cl (Figs 3A and 7G) [18]. Thus, HOCl-oxidised proteins may share the mechanism of the Th2-polarising effect with products (proteins and glycoproteins) excreted/secreted by parasitic worms, which were found to be ligands of the same endocytic receptors as HOCl-modified proteins, including MR [76–78] and SR-A [79]. Acting on APC these products inhibit IL-12 and enhance IL-10 production and, consequently, induce strongly Th2-polarised immune responses in vivo [76–79].

Improved immunogenicity of HOCl-modified proteins might be exploited in the development of more effective vaccines that, possibly, would not require co-administration of adjuvants. Currently, the research on the potential application of HOCl-oxidised proteins in anti-cancer vaccines is the most intensively pursued area and promising immunological and clinical results have been already reported [80,81].

## Supporting Information

S1 Fig One-day treatment with LPS, but not with OVA or OVA-Cl induces maturation of BM-DC, as indicated by up-regulated expression of MHC-II and CD40 molecules (A) and cytokine production (B). (TIF)

S2 Fig *S*. *aureus*- or zymosan-stimulated, lucigenin-enchanced chemiluminescence in WT and MPO-/- neutrophils. (TIF)

S3 Fig Titers of antigen-specific antibodies in sera of WT and MPO-/- mice immunized with: (A) HSA and zymosan depleted with TLR2 agonists or (B) OVA and heat-killed *S*. *aureus* or Pam3CSK4 lipopeptide. (TIF)

S4 Fig Roles of CD36 and SR-A as receptors for HOCl-oxidised proteins. (**A**, **B**) Effects of anti-CD36 CRF D-2712 mAb (A) and anti-SR-A 2F8 mAb (B) on AF-AcLDL, AF-OVA or AF-OVA-Cl uptake by BM-DC. (**C**) Effects of AcLDL or oxLDL on AF-OVA-Cl or pHr-OVA-Cl uptake by BM-DC or PEM isolated from WT, SR-A-/- and CD36-/- mice.
(TIF)

S5 Fig Comparison of fluorescently-labelled ligands uptake by BM-DC *vs*. PEM (TIF)

S6 Fig The role of LOX-1 as a receptor for HOCl-oxidised proteins. (**A**) A relationship between the magnitude of AF-OVA-Cl uptake and LOX-1 expression on BM-DC. (**B**) LOX-1 expression on LPS-pre-treated C57Bl/6 BM-DC.
(TIF)

S7 Fig Roles of SREC-I, RAGE and stabilin-1 as receptors for HOCl-oxidised proteins. (**A**) Binding of rSREC-I to proteins adsorbed to ELISA plates. (**B**) Expression of SREC-I on PEM. (**C**) Effects of anti-SREC-I Ab on AF-AcLDL uptake by BM-DC. (**D**) Binding of anti-RAGE Ab to BM-DC. (**E**) Binding of anti-stabilin-1 Ab to BM-DC.
(TIF)

S8 Fig The role of MR as a receptor for HOCl-oxidised proteins. (**A**, **B**) Dose dependent effects of mannan, CS (A) and DS (B) on rMR binding to plate-adsorbed OVA-Cl (A) or TFN-Cl (B). (**C**) Effects of 10 μg/ml or 100 μg/ml DS on rMR binding to wells coated with TFN-Cl vs. uncoated wells.
(TIF)

S9 Fig Titers of specific IgG in sera of intact mice or mice immunized with mouse TFN-Cl, human TFN or human TFN-Cl. C57BL/6 mice were injected *i*.*p*. twice with a 14-days interval with 20 μg of indicated antigens and titers of specific IgG in sera were determined 8 days after the second immunization.
(TIF)
