# Correction: Oxidation by Neutrophils-Derived HOCl Increases Immunogenicity of Proteins by Converting Them into Ligands of Several Endocytic Receptors Involved in Antigen Uptake by Dendritic Cells and Macrophages

**DOI:** 10.1371/journal.pone.0128612

**Published:** 2015-05-14

**Authors:** The PLOS ONE Staff

The following information is missing from the Funding section: The authors would like to express their gratitude to the Leading National Research Centre (KNOW) 2012–2017 for their financial support of the process of printing the article.

The publisher apologizes for the error.
