# Supplementary figures and images for: Oxidation by Neutrophils-Derived HOCl Increases Immunogenicity of Proteins by Converting Them into Ligands of Several Endocytic Receptors Involved in Antigen Uptake by Dendritic Cells and Macrophages
Source: PLoS One. 2015 Apr 7;10(4):e0123293. doi: 10.1371/journal.pone.0123293 (PMC4388828; doi:10.1371/journal.pone.0123293)

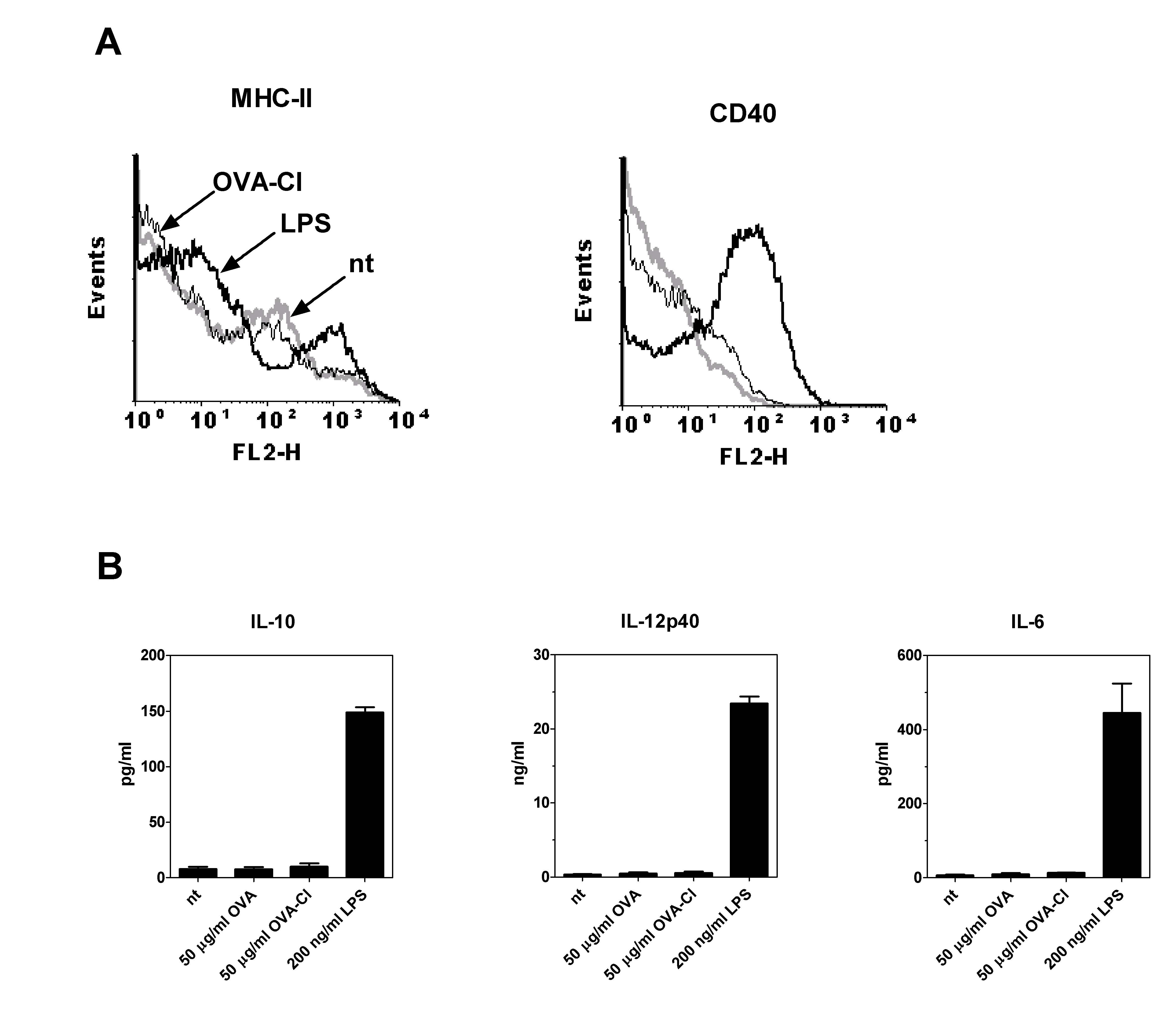

Supplement: S1 Fig — (TIF) [file pone.0123293.s001.tif]

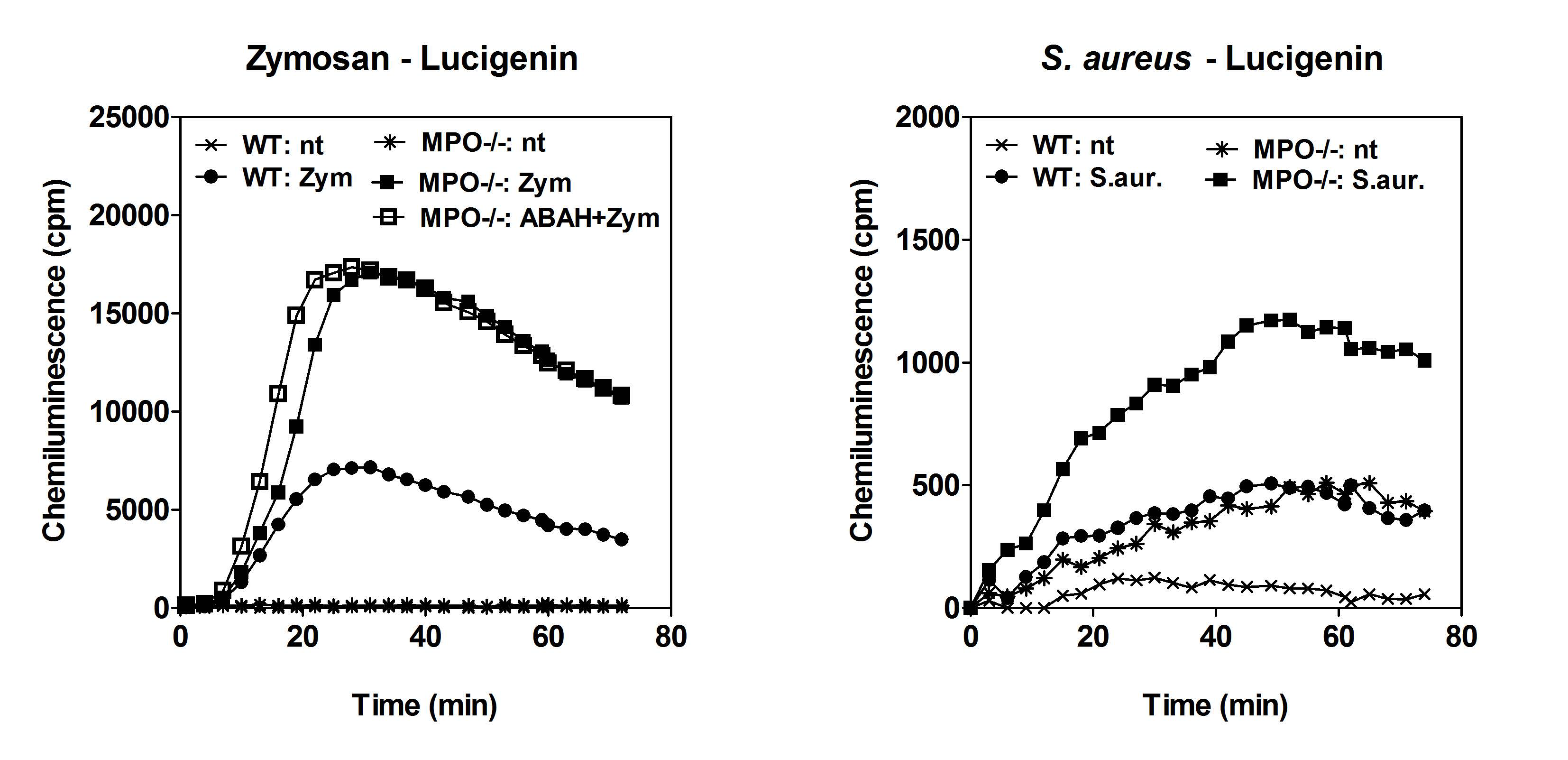

Supplement: S2 Fig — (TIF) [file pone.0123293.s002.tif]

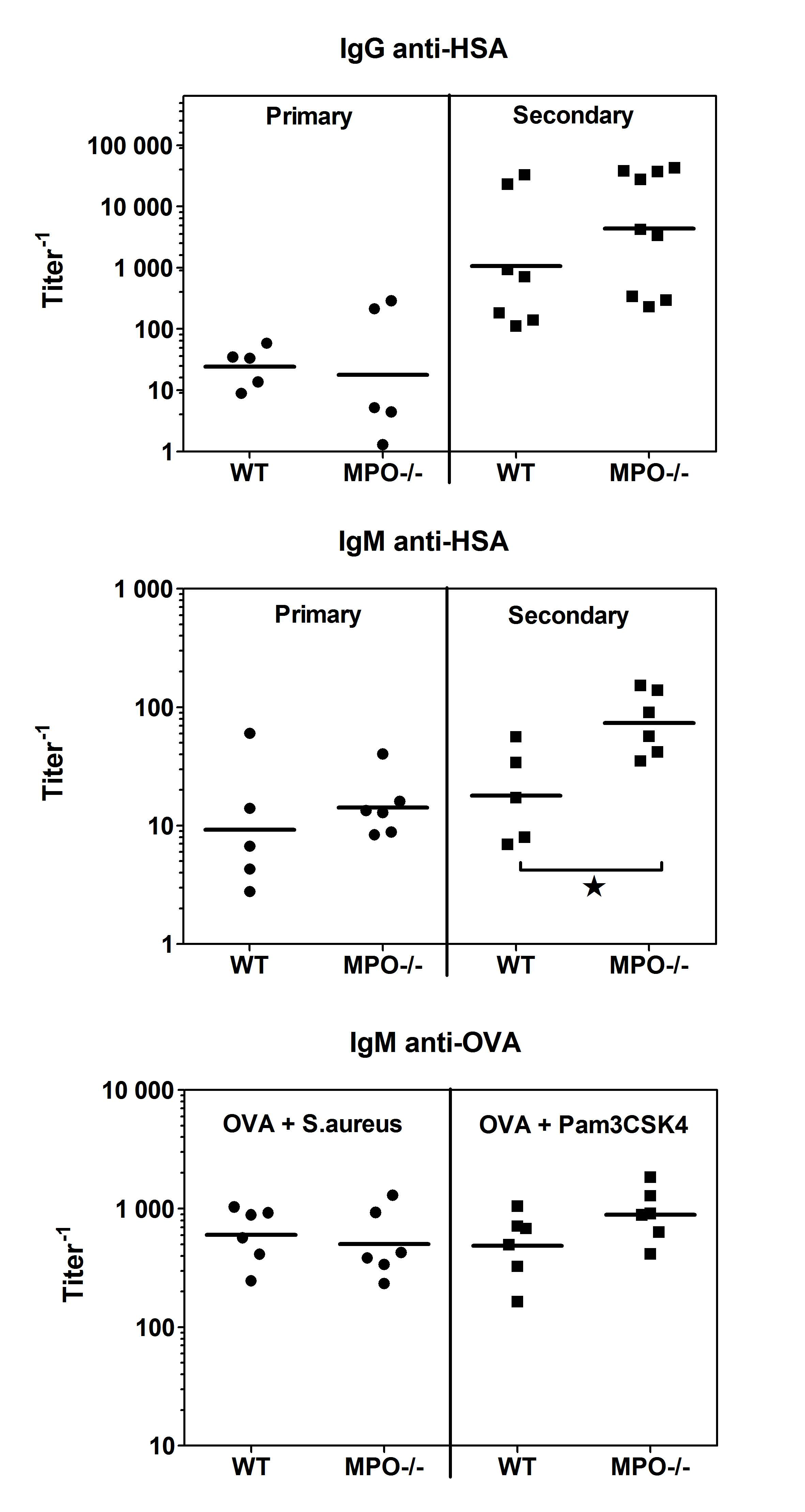

Supplement: S3 Fig — (TIF) [file pone.0123293.s003.tif]

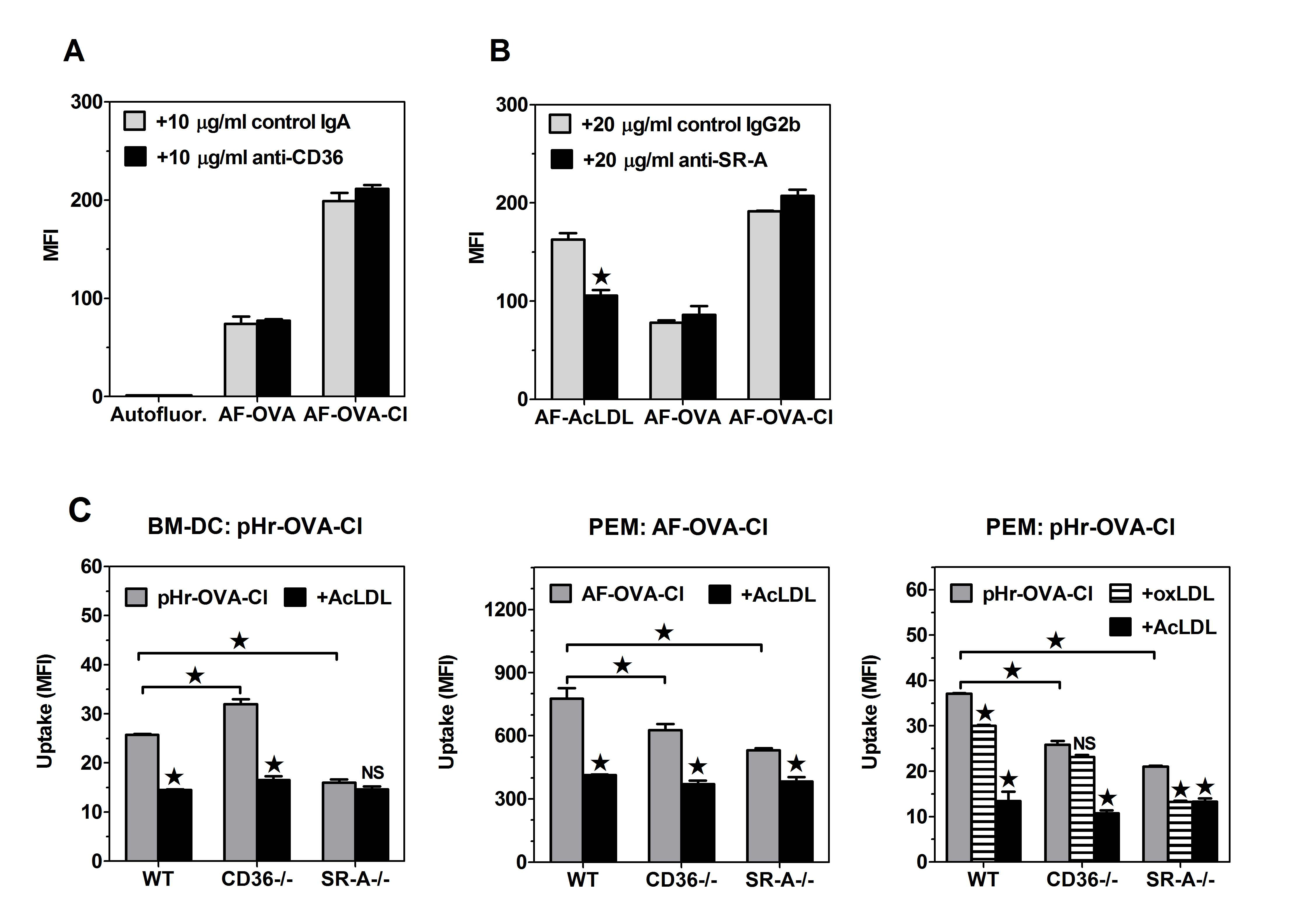

Supplement: S4 Fig — (A, B) Effects of anti-CD36 CRF D-2712 mAb (A) and anti-SR-A 2F8 mAb (B) on AF-AcLDL, AF-OVA or AF-OVA-Cl uptake by BM-DC. (C) Effects of AcLDL or oxLDL on AF-OVA-Cl or pHr-OVA-Cl uptake by BM-DC or PEM isolated from WT, SR-A-/- and CD36-/- mice. (TIF) [file pone.0123293.s004.tif]

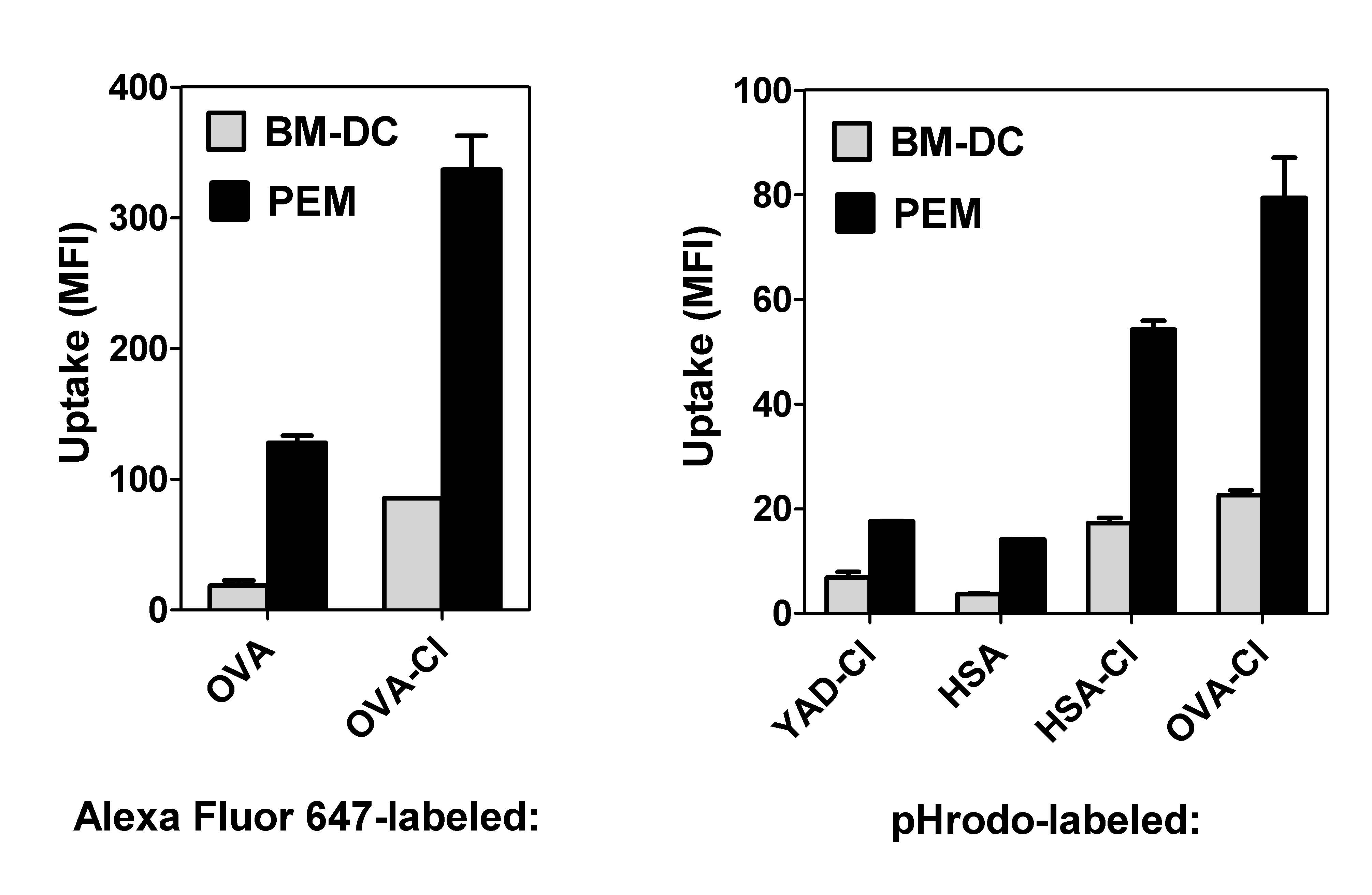

Supplement: S5 Fig — (TIF) [file pone.0123293.s005.tif]

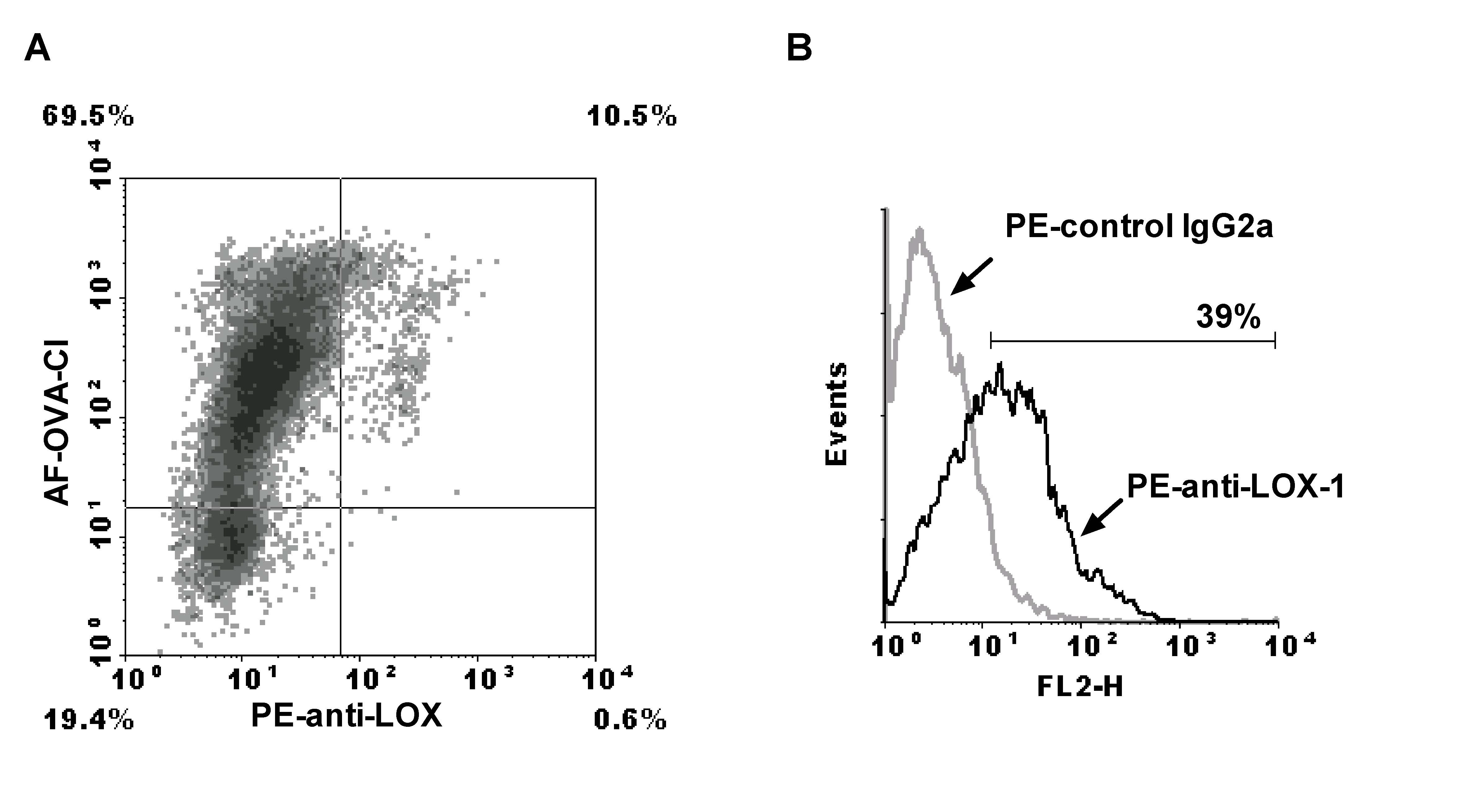

Supplement: S6 Fig — (A) A relationship between the magnitude of AF-OVA-Cl uptake and LOX-1 expression on BM-DC. (B) LOX-1 expression on LPS-pre-treated C57Bl/6 BM-DC. (TIF) [file pone.0123293.s006.tif]

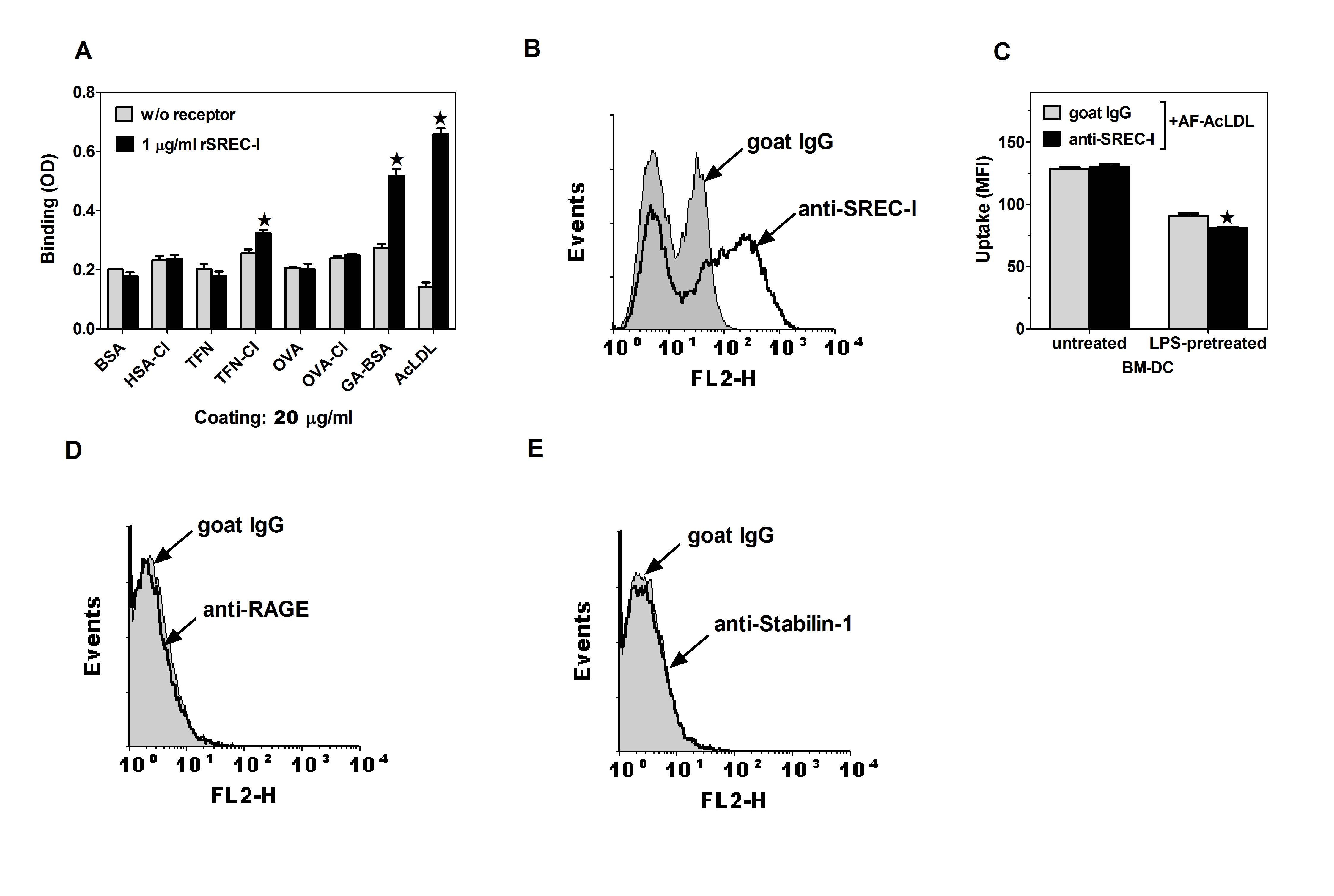

Supplement: S7 Fig — (A) Binding of rSREC-I to proteins adsorbed to ELISA plates. (B) Expression of SREC-I on PEM. (C) Effects of anti-SREC-I Ab on AF-AcLDL uptake by BM-DC. (D) Binding of anti-RAGE Ab to BM-DC. (E) Binding of anti-stabilin-1 Ab to BM-DC. (TIF) [file pone.0123293.s007.tif]

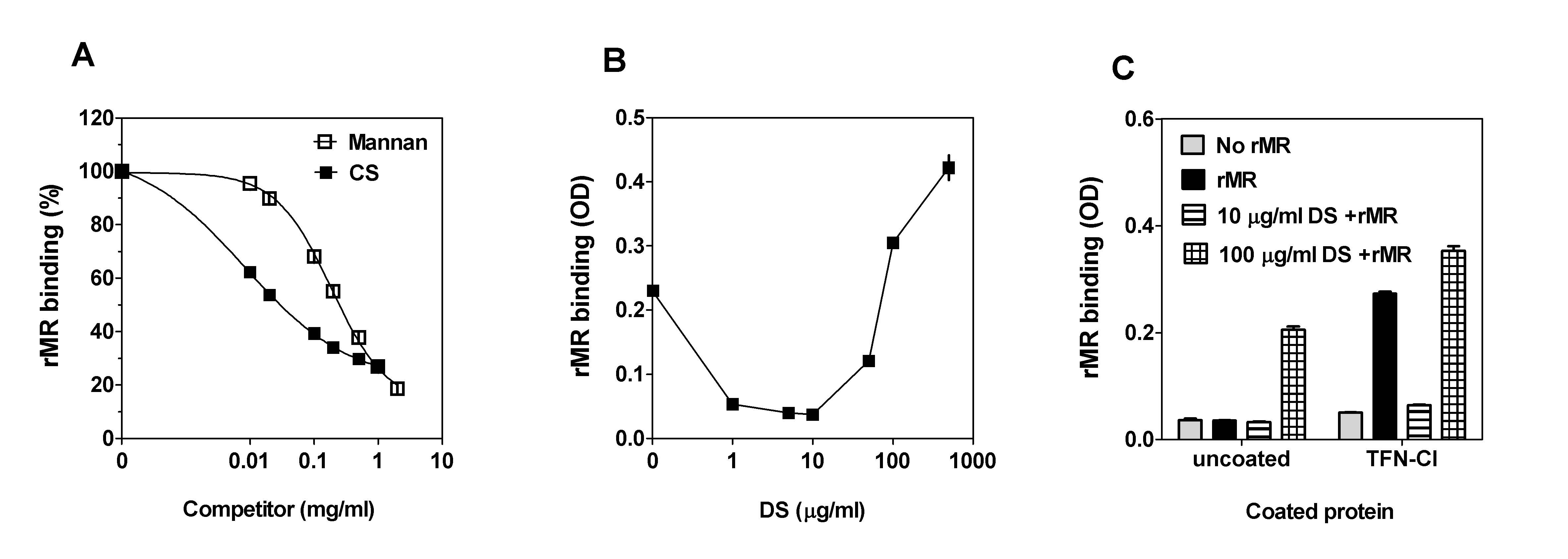

Supplement: S8 Fig — (A, B) Dose dependent effects of mannan, CS (A) and DS (B) on rMR binding to plate-adsorbed OVA-Cl (A) or TFN-Cl (B). (C) Effects of 10 μg/ml or 100 μg/ml DS on rMR binding to wells coated with TFN-Cl vs. uncoated wells. (TIF) [file pone.0123293.s008.tif]

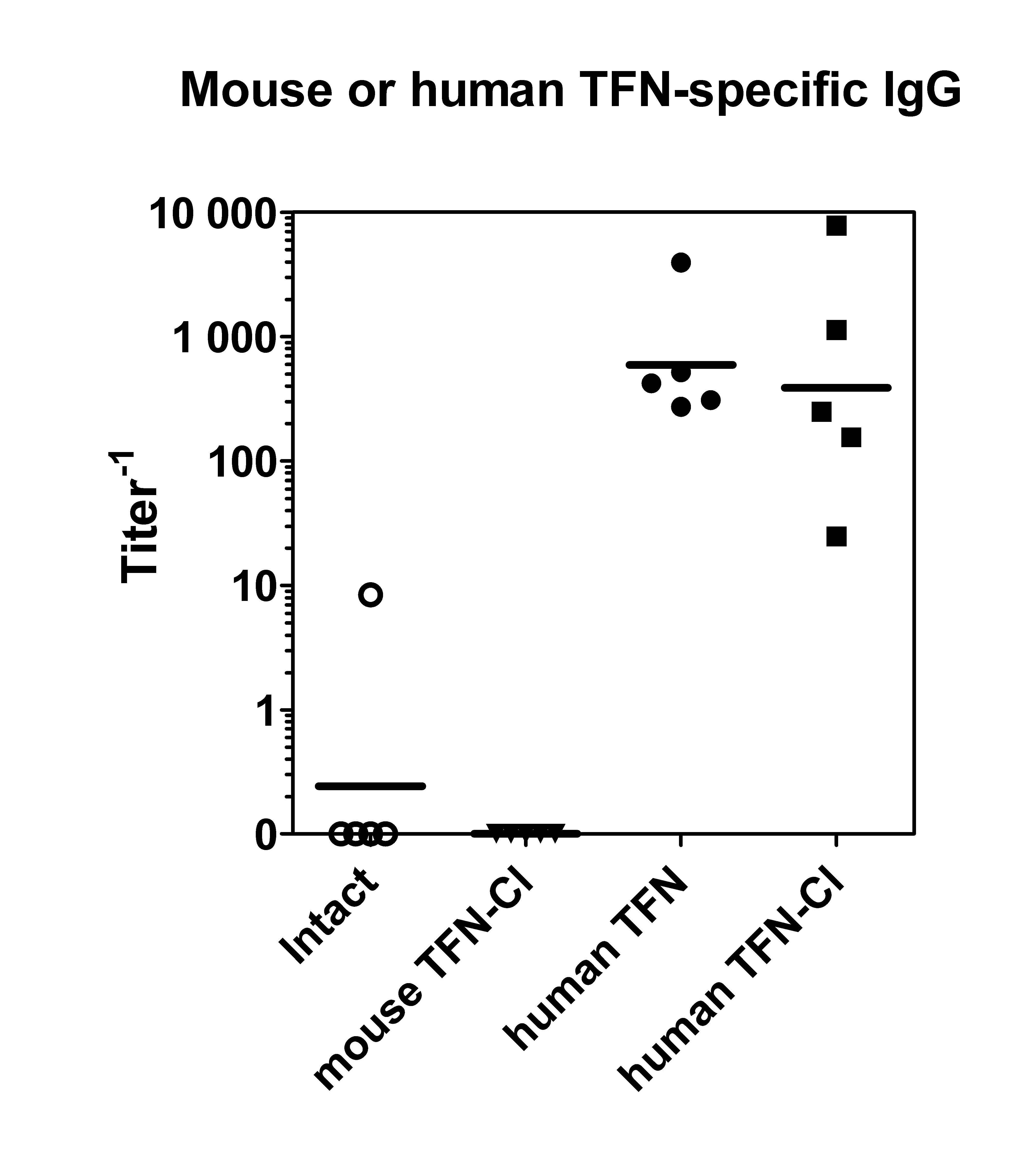

Supplement: S9 Fig — C57BL/6 mice were injected i.p. twice with a 14-days interval with 20 μg of indicated antigens and titers of specific IgG in sera were determined 8 days after the second immunization. (TIF) [file pone.0123293.s009.tif]
